# Transition-Metal-Catalyzed C–H/N–H Annulation Reactions between Imidazoles and Alkynes for the Synthesis of Fluorescent Benzimidazo[2,1‑*a*]Isoquinolines

**DOI:** 10.1021/acsomega.5c12541

**Published:** 2026-04-08

**Authors:** Gleiston G. Dias

**Affiliations:** Department of Chemistry, 28099 Pontifícia Universidade Católica Do Rio de Janeiro (PUC-Rio), 225 Marquês de São Vicente Street, Rio de Janeiro, Rio de Janeiro 22451-900, Brazil

## Abstract

This review summarizes recent advances in C–H/N–H annulation reactions between imidazoles and alkynes for the synthesis of imidazo­[2,1-*a*]­isoquinolines via transition-metal catalysis, including rhodium, ruthenium, cobalt, palladium, and nickel systems. These synthetic strategies enable the construction of highly conjugated fused heteropolycyclic aromatic frameworks. By providing an overview of the reported methodologies and discussing key regio- and stereochemical aspects, this article offers a concise guide to the synthesis and functionalization of these complex molecular architectures, with the aim of stimulating future studies toward applications in electronic and optoelectronic devices, which remain largely underexplored.

## Graphical abstract

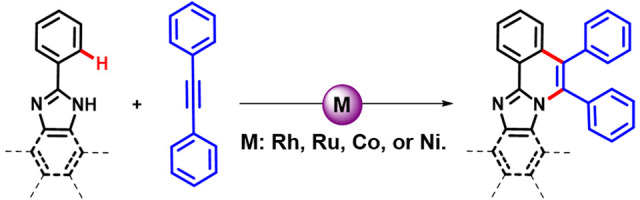

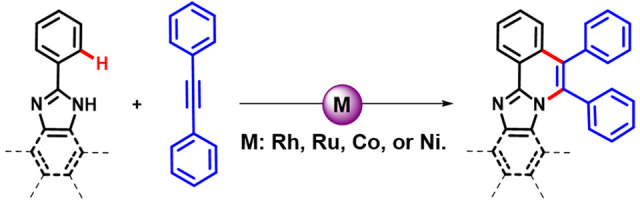

## 1 Introduction

The increasing interest in fluorescent organic molecules extends well beyond their visual beauty, which is often their initial allure. These compounds have found applications across a wide range of scientific fields, showcasing their versatility and impact in bioimaging,^1–4^ biological investigations,^5–8^ sensors for analyte detection,^9–12^ molecular logic gates,^13–15^ functional materials,^16–19^ organic light-emitting diodes (OLEDs),^20–22^ among many others. A fluorophore is typically designed as a molecule with a rigid, conjugated system, often consisting of fused aromatic rings. In this sense, polycyclic or heteropolycyclic aromatic compounds have been developed and synthesized as materials for organic electronic and optical applications.

Numerous fluorescent cores have been investigated, such as anthracene,^23–25^ phenazine,^26^ quinoline,^27–30^ acridine,^31^ BODIPy,^32–34^ 2,1,3-benzothiadiazole (BTDs),^35^ coumarin,^36–38^ fluorescein,^39–41^ rhodamine,^42–45^ pyrene,^46–48^ pyrazine,^49,50^ and others. Another example is the imidazole, a five-membered heterocyclic compound with two nitrogen atoms at nonadjacent positions in the ring, also known as 1,3-diazole.^51^ A wide range of imidazole derivatives with diverse applications has been developed by exploring structural diversity and incorporating molecular groups with various functionalities. Examples of these applications include fluorescent sensors for analyte detection,^52^ solar cells,^53^ medicinal chemistry,^54^ materials,^55,56^ and OLEDs.^57^

The advent of metal-catalyzed C–H activation processes has provided an innovative pathway for synthesizing new fluorescent molecular scaffolds or modifying traditional ones.^58–61^ This specific application focuses on expanding the π-conjugated system, primarily by constructing fused aromatic rings through C–H annulation reactions.

This review discusses advances in modifying imidazoles through metal-catalyzed C–H/N–H annulation, focusing on creating or modifying highly conjugated fluorescent polycyclic compounds. Particular emphasis is placed on using diphenyl alkynes as a reagent for annulation, as it leads to a more conjugated system due to incorporating two phenyl groups into the molecular structure.

## 2 Synthesis of Imidazole

Strong fluorescence emission in imidazoles is typically observed when 1*H*-imidazole (**1**) is fused with other aromatic systems, forming highly conjugated and planar structures. This results in derivative compounds such as 1*H*-benzo­[*d*]­imidazole (**2**), 3*H*-naphtho­[1,2-*d*]­imidazole (**3**), 1*H*-phenanthro­[9,10-*d*]­imidazole (**4**), 9*H*-pyreno­[4,5-*d*]­imidazole (**5**), and so on (Scheme 1).

**Scheme 1:**
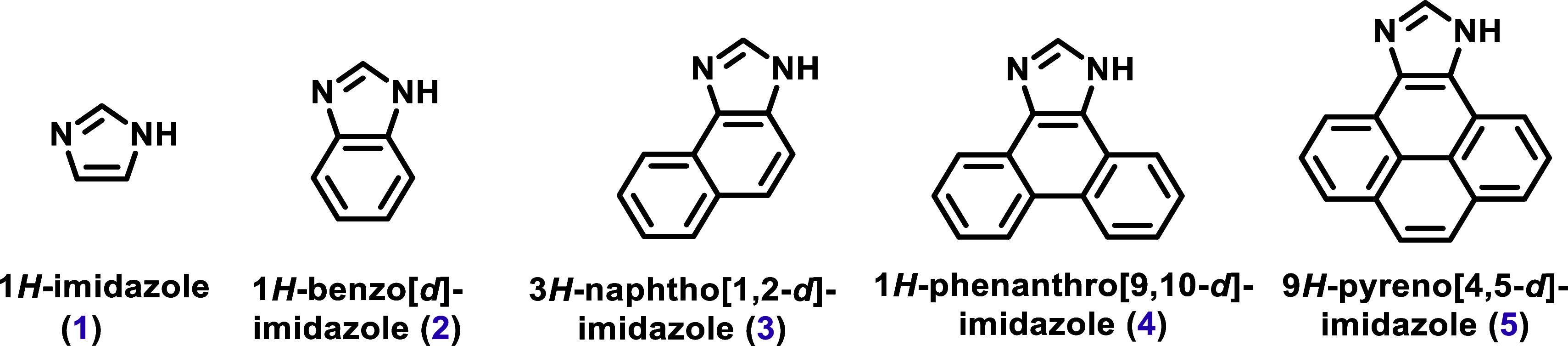
1*H*-Imidazole (**1**) and its Derivatives 1*H*-Benzo­[*d*]­Imidazole (**2**), 3*H*-Naphtho­[1,2-*d*]­Imidazole (**3**), 1*H*-Phenanthro­[9,10-*d*]­Imidazole (**4**), 9*H*-Pyreno­[4,5-*d*]­Imidazole (**5**).

Guo et al. investigated the optical properties of 2,4,5-triphenyl-1*H*-imidazole (**6**) and 2-phenyl-1*H*-phenanthro­[9,10-*d*]­imidazole (**7**) using density functional theory (DFT).^62^ They concluded that the phenyl rings in compound **6** are out of the imidazole plane due to steric effects (Scheme 2). This deviation from planarity reduces the conjugation of the π-system. In contrast, in compound **7**, both phenyl rings are fused to the imidazole, enhancing planarity and rigidity. The consequence of this structural restriction is the expansion of π-system conjugation, leading to a decrease in the energy gap between the HOMO and LUMO orbitals, as observed for compound **7** compared to 2,4,5-triphenyl-1*H*-imidazole (**6**). These electronic features led to an increased fluorescence quantum yield and caused a bathochromic shift in absorption and emission of compound **7** relative to compound **6**. These studies demonstrate the importance of rigid, conjugated rings fused to imidazole for a molecule to exhibit fluorescence.

**Scheme 2:**
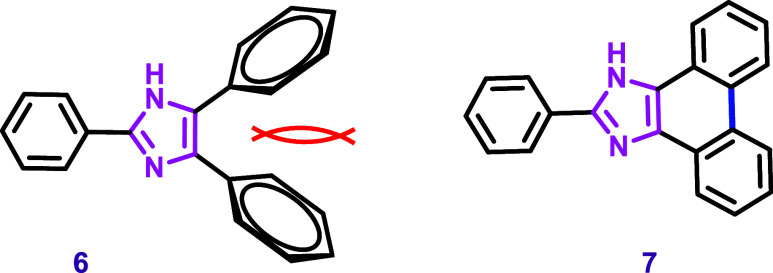
Repulsion between Phenyl Rings Reduces the Planarity and π-Conjugation in 2,4,5-Triphenyl Imidazole (**6**), Whereas the Fused Phenyl Rings in 9,10-Phenanthroimidazole (**7**) Enhance Planarity and Conjugation, as Described by Guo et al.^62^

There are several synthetic approaches to prepare imidazoles;^63–72^ however, one of the most commonly used strategies to synthesize imidazole-fused aromatic systems involves a reaction between 1,2-dicarbonyl compounds (**8**) and aldehyde derivatives (**9**) in the presence of a nitrogen source, such as ammonium acetate (**10**) (Scheme 3).

**Scheme 3:**
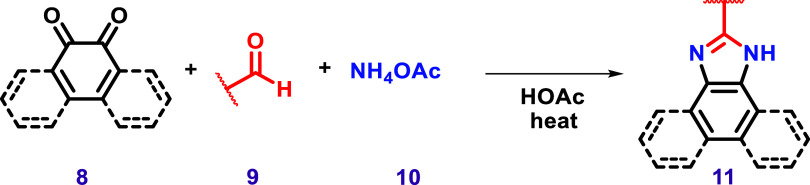
General Representation of the Synthesis of Imidazoles via a Multicomponent Reaction between a 1,2-Dicarbonyl (**8**) Compound and an Aldehyde (**9**) in the Presence of Ammonium Acetate (**10**).

Beyond the synthesis of imidazoles through classical condensations, the advent of cross-coupling reactions has opened a pathway for the sequential transformation of this heterocycle. In this context, the ability to form C–C or C–N bonds at sp^2^ carbons, typically mediated by a palladium catalyst, provides a route for designing new fluorophores. Among these methodologies, the coupling reactions of Sonogashira, Suzuki, Heck, Stille, and Buchwald–Hartwig stand out. These prominent synthetic approaches are known for efficiently modifying aromatic systems.

Although the coupling reactions mentioned have been proven strategic in developing fluorophores, the requirement for preinstalled molecular groups (such as halogen or boronic acid) on the reagents can sometimes limit synthetic possibilities. These limitations are often associated with side reactions, the use of expensive chemicals, and multiple steps that generate waste. In this context, C–H activation has emerged as an alternative and complementary approach in designing and modifying fluorophores, including imidazoles.^58–61^

## 3 C–H Activation in Imidazole

Condensation and coupling reactions may be broadly understood as Functional Group Interconversion (FGI), as one functional group is replaced by another through bond cleavage, migration, and substitution. Such transformations are possible once these bonds are in a highly energy state, known as activated bonds.^73^ On the other hand, nonacidic C–H bonds, the most prevalent functional group in organic chemistry, are considered deactivated due to their high kinetic and thermodynamic stability, resulting in their low reactivity.^74^

The modification of a low-reactivity C–H bond, induced by a transition metal (C–metal intermediate), without prior functionalization (oxidation), is called C–H activation.^75^ This synthetic approach allows the direct installation of a functional group onto an inert C–H bond, unlike classical condensation and coupling reactions that rely on FGI.^76^ Some C–H activation examples include arylation,^77^ halogenation,^78^ amination,^79^ alkylation,^80^ and oxygenation.^81^

Usually, in C–H bond activation, a directing group (DG) with lone electron pairs coordinates to the transition metal of the catalyst, followed by a cyclometalation. Subsequently, a functional group is inserted to promote the conversion of C–H to a C–FG.^82^ Several groups have been used as directing groups in C–H activation. Some examples include pyridine,^83^ triazoles,^84^ pyrazoles,^85^ amides,^86^ carboxylic acids,^87^ ketones,^88^ hydrazones,^89^ and others.^90^

Similarly, the nitrogen in imidazole can also serve as a DG to promote C–H activation.^91^ In this context, the N–H group of imidazole can be metalated during an organometallic catalytic reaction, acting as a DG to activate the *ortho* position of an aryl group located at position 2 of the imidazole (Scheme 4A). For instance, Kamal et al. described a regioselective aryl C–H hydroxylation of 2-aryl-benzimidazoles using a Pd­(OAc)_2_/oxone/Cs_2_CO_3_ catalytic system (Scheme 4B).^92^

**Scheme 4:**
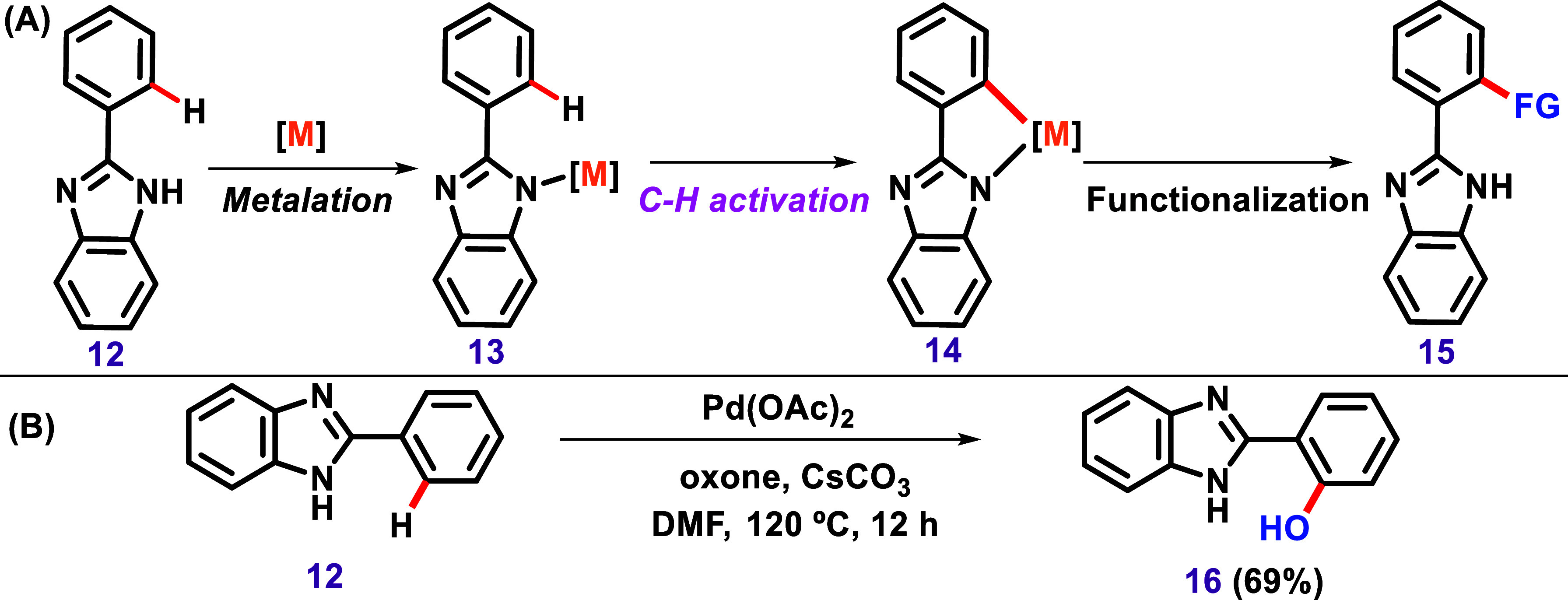
(A) General Representation of N–H Metalation of the Imidazole Followed by C–H Activation at the *Ortho*-Position and Insertion of a Functional Group. (B) C–H Hydroxylation of 2-Aryl Benzimidazoles Described by Kamal et al.^92^

One approach of C–H activation of imidazole involves annulation reactions in which the nitrogen of the imidazole acts as the DG, and the *ortho*-position of the 2-aryl group forms a new cycle with the imidazole core (Scheme 5A), followed by annulation with a functional group. This protocol, commonly referred to as C–H/N–H annulation, provides an effective strategy for modifying imidazole, resulting in a more rigid structure.

**Scheme 5:**
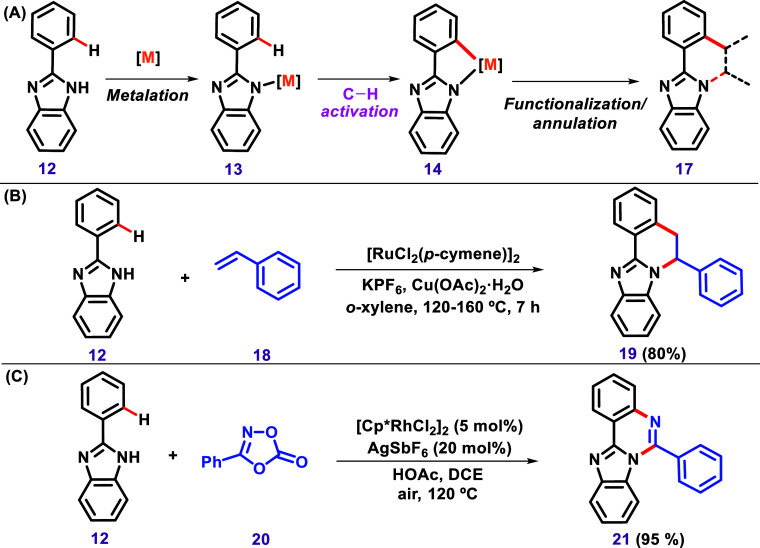
(A) General Representation of C–H/N–H Annulation in Imidazole (B) C–H/N–H Annulation of 2-Aryl Benzimidazole (**12**) and Styrene (**18**) via Ru­(II)-Catalyzed [4 + 2] Annulation to Afford Benzoimidazo­[2,1-*a*]­Isoquinolines (**19**) Described by Dhole et al.^93^ (C) Rh-Catalyzed Reaction between 2-Aryl Imidazole (**12**) and 3-Phenyl-1,4,2-Dioxazol-5-Ones (**20**) to Form 5-Arylimidazo­[1,2-*c*]­Quinazolines (**21**) via a Sequential *ortho*-C–H Bond Amidation and Cyclization Described by Wu et al.^94^

For instance, Dhole and Sun described the reaction of 2-aryl-benzimidazole (**12**) and styrene (**18**) via Ru­(II)-catalyzed [4 + 2] annulation to afford benzimidazo­[2,1-*a*]­isoquinolines (**19**). Mechanistic studies led the authors to conclude that the reaction involves sequential C–C/C–N bond formation (Scheme 5B).^93^ Wu et al. proposed a Rh-catalyzed reaction between 2-aryl imidazoles (**12**) and 3-phenyl-1,4,2-dioxazol-5-ones (**20**) to achieve 5-arylimidazo­[1,2-*c*]­quinazolines (**21**) via a sequential *ortho*-C–H bond amidation and cyclization (Scheme 5C).^94^ Other similar synthetic approaches have been developed to synthesize additional heterocycles based on the C–H/N–H annulation of imidazole.^95,96^

Since the basic structural framework of a fluorescent compound typically involves conjugated cyclic and rigid systems, increasing its conjugation may lead to modulation of its optical properties. For instance, a more rigid system may display bathochromic shifts and increase the quantum yield. In this context, C–H/N–H annulation of imidazole can modify its electronic density and, consequently, its optical properties. This phenomenon can primarily be attributed to the junction of the aryl group with the imidazole, which enhances the planarity of the compound and/or insertion of groups capable of improving electronic conjugation.

For instance, the reaction described in Scheme 5B shows a restriction in the rotation of the phenyl group attached to the imidazole ring. In the second example, in Scheme 5C, there is not only a rotation restriction but also an increase in the conjugation of the π-system.

The C–H/N–H arylation of imidazole to afford phenanthridines derivatives (**24**) (Scheme 6) is an approach that addresses both structural effects: restriction of the 2-aryl and an increase in conjugation. This reaction can be accomplished by using 1,2-dihaloarenes in palladium-catalyzed tandem C–H/N–H arylation, as described by Yan et al. (Scheme 6A).^97^ The authors synthesized compound **26** in 96% yield using 1,2-dibromobenzene (**25**). Chen et al. described a palladium-catalyzed synthesis of benzimidazole-fused phenanthridines from *o*-dibromoarenes and 2-aryl benzimidazoles via an intermolecular C–H arylation of 2-aryl benzimidazoles followed by an intramolecular *N*-arylation reaction.^98^ Liu et al. described one-pot synthesis through a cascade palladium-catalyzed *N*-arylation and intramolecular C–H coupling to synthesize the product.^99^ Another methodology to achieve compound **26** employs iodobenzene (**27**), as reported by Zhao et al., instead of 1,2-dibromobenzene (**25**).^100^ In 2022, Geng et al. described a new reaction for a tandem Pd/Cu-catalyzed intermolecular cross-coupling cascade between *o*-bromobenzoic acids and 2-(2-bromoaryl)-1*H*-benzo­[*d*]­imidazoles in a controlled and regioselective manner to afford compound **26** (Scheme 6B).^101^

**Scheme 6:**
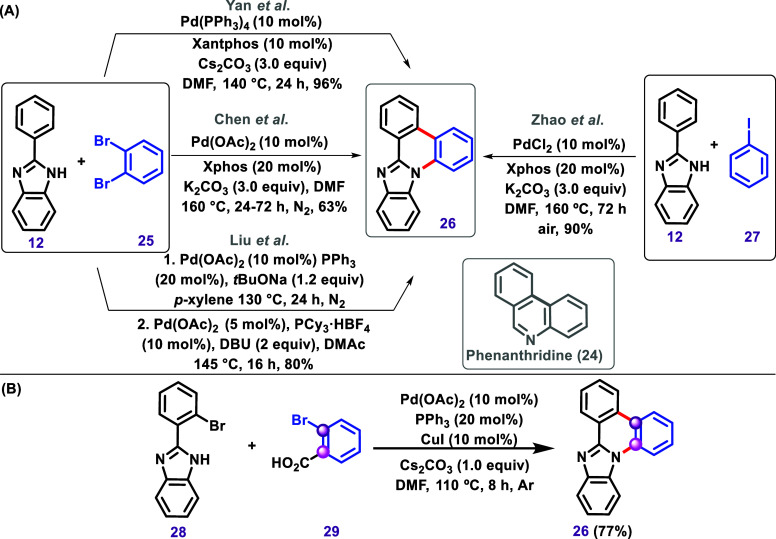
(A) C–H/N–H Annulation via Insertion of a benzene Using 1,2-Dibromobenzene (**25**) Described by Yan et al.,^97^ Chen et al.,^98^ and Liu et al.^99^ and Using Iodobenzene, Described by Zhao et al.^100^ (B) a Tandem Pd/Cu-Catalyzed Intermolecular Cross-Coupling Cascade between *o*-Bromobenzoic Acids and 2-(2-Bromoaryl)-1*H*-Benzo­[*d*]­Imidazoles Described by Geng et al.^101^

The heterocycle **26** may also be obtained by using 2-([1,1′-biphenyl]-2-yl)-1*H*-benzo­[*d*]­imidazole (**30**), in other words, a substrate bearing a phenyl group already attached to the imidazole, as reported by Bera et al. in 2019 ^102^ and 2021 ^103^ and also for Shi et al. (Scheme 7).^104^ However, it is essential to note that none of these cases involved the use of an organometallic catalyst; therefore, these reactions are not classified as C–H bond activation. Bera et al. described^102^ a C–N coupling reaction using iodine­(III) reagent PhI­(OCOCF_3_)_2_ (PIFA) via the formation of an antiaromatic endocyclic nitrenium ion **31** (Scheme 7A). Later, the same research group reported a direct photochemical dehydrogenative C–N coupling carried out under ∼350 nm of irradiation via ε-hydrogen abstraction, forming the 1,6-diradical **32** (Scheme 7B).^103^ Shi et al. developed an electrochemical intramolecular C–H amination of 2-([1,1′-biphenyl]-2-yl)-1*H*-benzo­[*d*]­imidazole (**30**), mediated by tris­(4-bromophenyl)­amine (**33**) under oxidant- and metal-free conditions (Scheme 7C).^104^

**Scheme 7:**
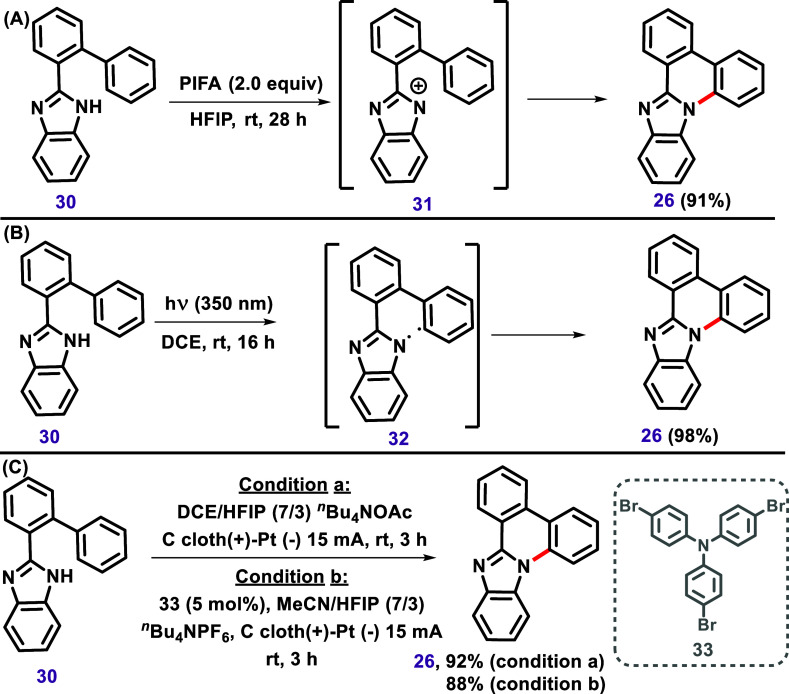
Cyclization of 2-([1,1′-Biphenyl]-2-yl)-1*H*-Benzo­[*d*]­Imidazole (**30**) to Afford Compound **26** Described by (A) Bera et al. In 2019,^102^ (B) in 2021,^103^ (C) and also by Shi et al. in 2021.^104^

Another approach to modifying imidazole to increase its rigidity and conjugation involves the insertion of a styrene unit to afford 6-phenylbenzo­[4,5]­imidazo­[2,1-*a*]­isoquinoline derivatives using different strategies (Scheme 8). For instance, Gang et al.^105^ and Zhang et al.^106^ (Scheme 8A,B) developed protocols using phenylacetylene under copper catalysis to afford compounds **36** and **38**, respectively, based on the Sonogashira reaction followed by intramolecular hydroamination. Miao et al.^107^ proposed a sequential α-arylation of carbonyl and deacylation catalyzed by CuI (Scheme 8C) using 1,3-diketones to afford **40**, similarly described by Yang et al.^108^ Diep et al.,^109^ also using 1,3-diketones, applied microwave irradiation in DMF in the presence of a catalytic amount of recyclable Fe_3_O_4_@SiO_2_@MOF-199 to afford the product **42** (Scheme 8D). Recently, Lee et al. described a transition-metal-free approach to synthesize **42** via KO^*t*^Bu/DMSO system (Scheme 8E).^110^ Although these methodologies have proven efficient, it is essential to note that none involve the activation of C–H bonds.

**Scheme 8:**
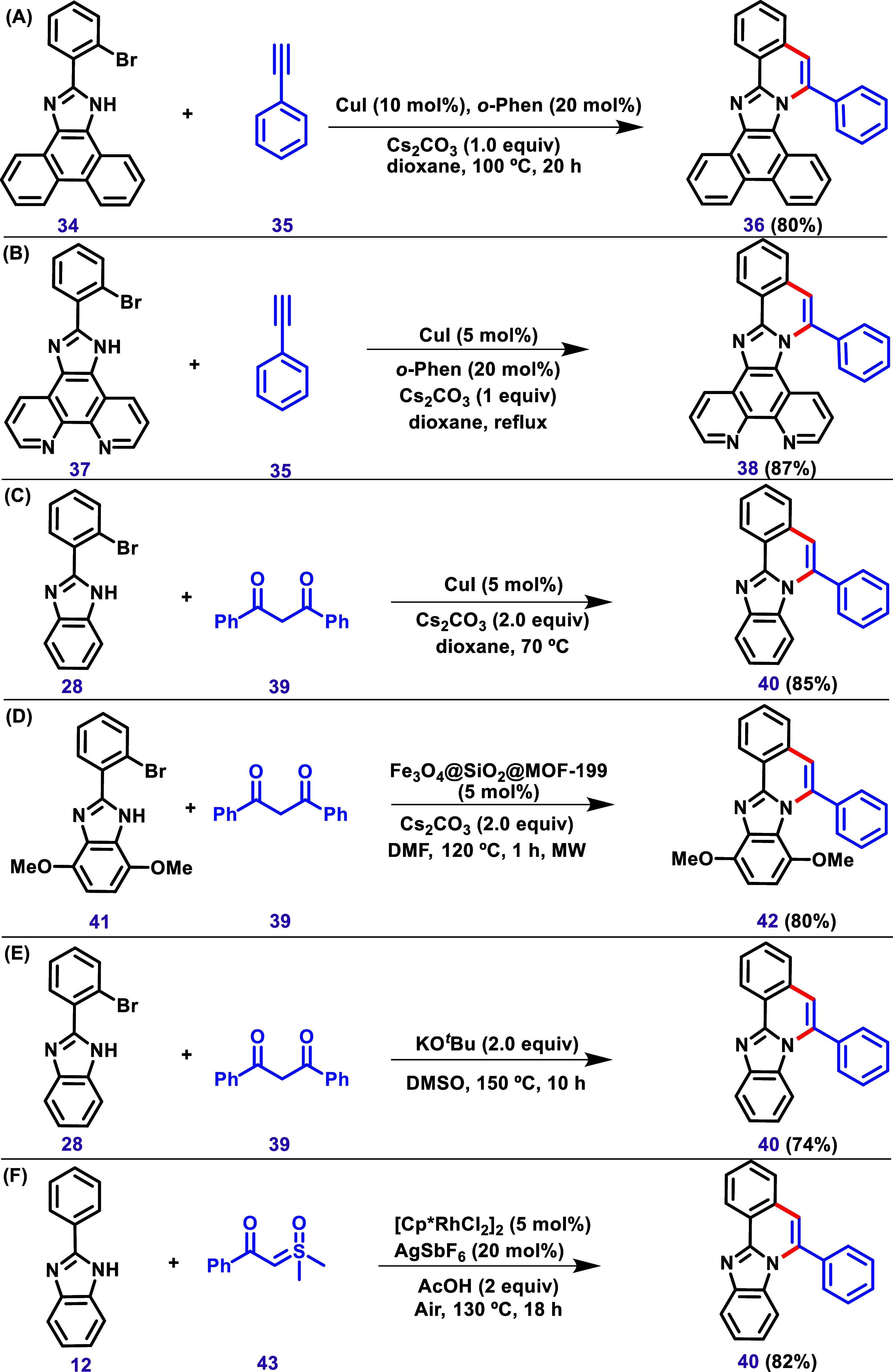
Synthesis of 6-Phenylbenzo­[4,5]­Imidazo­[2,1-*a*]­Isoquinoline (**36**) Derivatives Using Phenyl Acetylene (**35**) Described by (A) Gang et al.,^105^ (B) Zhang et al.,^106^ and the Similar Product **40** and **42**, Respectively, Using 1,3-Diketones (**39**) Described by (C) Miao et al.,^107^ (D) Diep et al.,^109^ and (E) Lee et al.^110^ (F) Synthesis of 6-Phenylbenzo­[4,5]­Imidazo­[2,1-*a*]­Isoquinoline (40) via a Rh-Catalyzed Annulation between 2-Aryl-1*H*-Benzo­[*d*]­Imidazole (**12**) and α-Aroyl Sulfoxonium Ylides (**43**) Described by Yang et al.^111^

One example of obtaining product **40** without the prefunctionalization with bromine (C–H activation) was described by Yang et al.^111^ via an Rh-catalyzed annulation between 2-aryl-1*H*-benzo­[*d*]­imidazoles and α-aroyl sulfoxonium ylides (Scheme 8F). According to the authors, this reaction occurs via activation, followed by sequential *ortho*-C–H functionalization and cyclization.

The synthesis of a target molecule in a multistep reaction using a single reaction vessel is referred to as a one-pot reaction. This approach can reduce chemical waste, save time, and simplify synthetic procedures, making it an efficient and environmentally sustainable process.^112^

In this sense, Okamoto et al. described a one-pot reaction involving 2-bromophenylaldehyde (**44**), phenylacetylene (**35**), and 1,2-phenylenediamine (**45**) to afford benzimidazo­[2,1-*a*]­isoquinoline (**40**) under microwave assistance (Scheme 9A).^113^ In this tandem process, Sonogashira coupling, 5-endo cyclization, oxidative aromatization, and 6-endo cyclization were described in one synthesis step. Rustagi et al. described an Ag­(I)-catalyzed cascade approach to achieve substituted fused benzimidazo­[2,1-*a*]­isoquinolines in water (Scheme 9B).^114^ In this protocol, the authors used 2-(phenylethynyl)­benzaldehyde (**46**) instead of generating it in situ, as described by Okamoto et al.^113^ Gvozdev et al. described a cyclization between *o*-alkynyl benzaldehyde and *o*-diamino benzenes in DMSO and NH_4_Br to afford 11-arylmethylidene-11*H*-isoindolo­[2,1-*a*]-benzimidazoles via a 5-*exo*-dig ring closure (Scheme 9C).^115^ Another example of a one-pot reaction was described by Peng et al. via a nucleophilic addition of benzimidazoles to alkynyl bromides via palladium-catalyzed intramolecular C–H vinylation (Scheme 9D).^116^ In this protocol, unlike the others, the authors used bromoacetylene. Kethe et al. described the synthesis of the regioisomer **50** via the induction of cyclodehydration using CF_3_SO_3_H as a superacid system (Scheme 9E).^117^

**Scheme 9:**
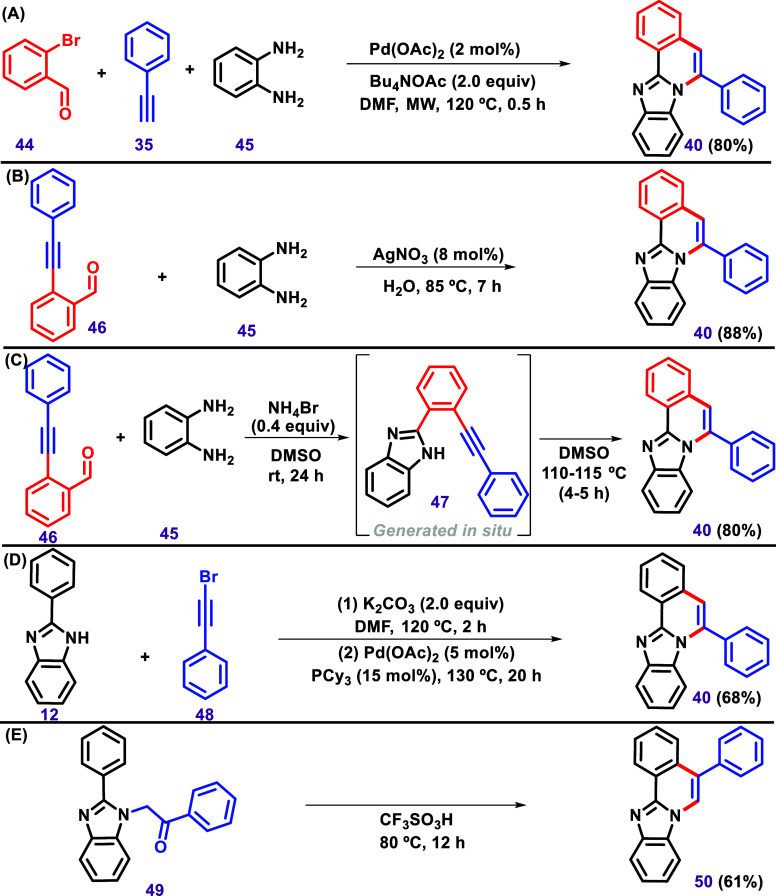
Synthesis of 6-Phenylbenzo­[4,5]­Imidazo­[2,1-*a*]­Isoquinoline via (A) One-Pot Reaction, (b)­Ag­(I)-Catalyzed Cascade Approach^114^ (c) NH_4_Br-DMSO^115^ System, and (d) Nucleophilic Addition of Benzimidazoles to Alkynyl Bromides via Palladium-Catalyzed Intramolecular C–H Vinylation^116^ (e) Synthesis of 5-Phenylbenzo­[4,5]­Imidazo­[2,1-*a*]­Isoquinoline via Induction of Cyclodehydration Using the CF_3_SO_3_H as a Superacid System.^117^

In this context, C–H activation of imidazole toward annulation, which increases the aromatic system, can be considered an essential strategy in the design of fluorophores. Although several protocols involving C–H/N–H annulation of imidazole have been published, the use of disubstituted alkynes has stood out, likely due to the ability to achieve highly conjugated products, as exemplified by the case involving benzimidazoles (Scheme 10). The product obtained in this reaction can be understood as a fusion of a benzimidazole with an isoquinoline,^118^ thus named benzo­[4,5]­imidazo­[2,1-*a*]­isoquinoline.^119^

**Scheme 10:**
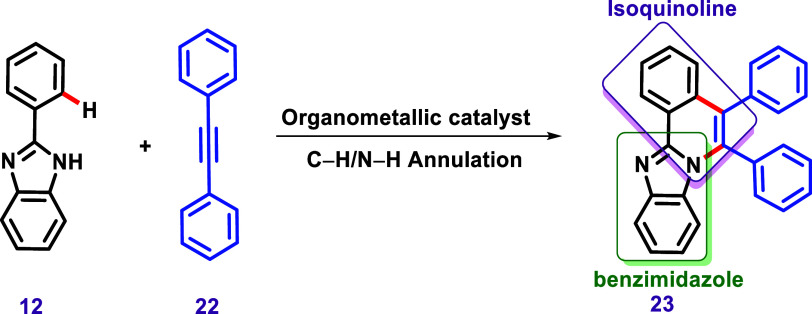
A General Example of C–H/N–H Annulation Using the 2-Phenyl Benzimidazole (**12**), Illustrating the Formation of Fused Isoquinoline and Benzimidazole Groups, Resulting in the Benzo­[4,5]­Imidazo­[2,1-*a*]­Isoquinoline Heterocycle.

## 4 Stereochemical and Synthetic Strategies on C–H/N–H of Imidazole

Before discussing imidazole C–H/N–H annulation, it is essential to consider certain stereochemical aspects, as the substitution pattern of these heterocycles can result in various isomers.

Imidazole can exist in solution as two isomeric forms due to the equilibrium of the proton from N–H between the two nitrogen atoms (Scheme 11). Therefore, these forms may not be equivalent depending on the position of a substituent in the imidazole relative to N–H. As a result, different isomers can be obtained from the C–H/N–H annulation.

**Scheme 11:**
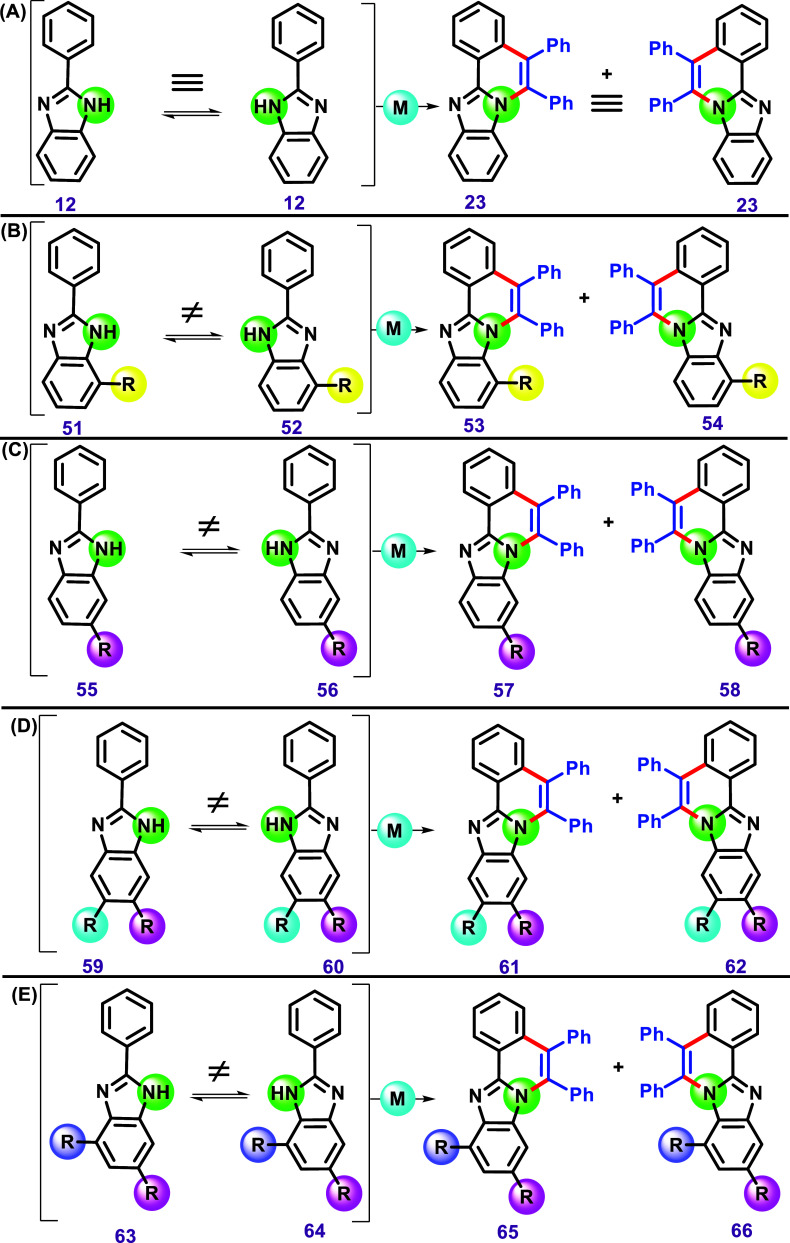
(A) A Generic Representation of the imidazole Proton Equilibrium, Resulting in the Formation of Two Identical Products. (B) A Representation of a Substituent Located at the 7-Position of the 2-Phenyl-1*H*-Benzo­[*d*]­Imidazole, Leading to the Formation of Two Isomers. (C) A Similar Result Occurs in the Case of a Substituent at the 6-Position, (D) the 5,6-Positions, and (E) the 4,6-Positions.

In the case of the nonsubstituted symmetrical compound **12**, both tautomeric forms are identical. Therefore, the C–H/N–H annulation using diphenylacetylenes leads to the single compound **23** (Scheme 11A). On the other hand, the proton equilibrium in a molecule bearing any substituent pattern on the aromatic group fused to the imidazole may result in a nonsymmetrical imidazole, meaning that two compounds, **51** and **52**, may exist in equilibrium (Scheme 11B). Consequently, the C–H/N–H annulation may result in two isomeric products, **53** and **54**. A similar case for isomer pairs **55** and **56** can be described, resulting in products **57** and **58** (Scheme 11C). Considering two different substituents on the benzimidazole ring (Scheme 11D,E), two isomers, **61**/**62** and **65**/**66**, can also be obtained in each case.

For our stereochemical analysis, we will now consider only a single substitution at the *ortho*, *meta*, and para positions of a phenyl ring attached to a benzimidazole, along with the proton equilibrium of imidazole and the free rotation of the substituted phenyl ring. As a result of the proton equilibrium and the free rotation of the phenyl ring, compound **67**, bearing an *ortho*-substituent, in a C–H/N–H annulation can lead to a single product (Scheme 12A). A similar result may be obtained for a substrate bearing a *para*-substituent (Scheme 12B). However, a substrate with a substituent in the *meta* position may present a different outcome (Scheme 12C). A pair of isomeric products can be obtained when this substrate undergoes a C–H/N–H annulation. Considering substituents in *ortho* and *para* positions, only one isomer is possible (Scheme 12D), similar to the case of substituents in the *ortho* or *para* positions separately (Scheme 12A,B).

**Scheme 12:**
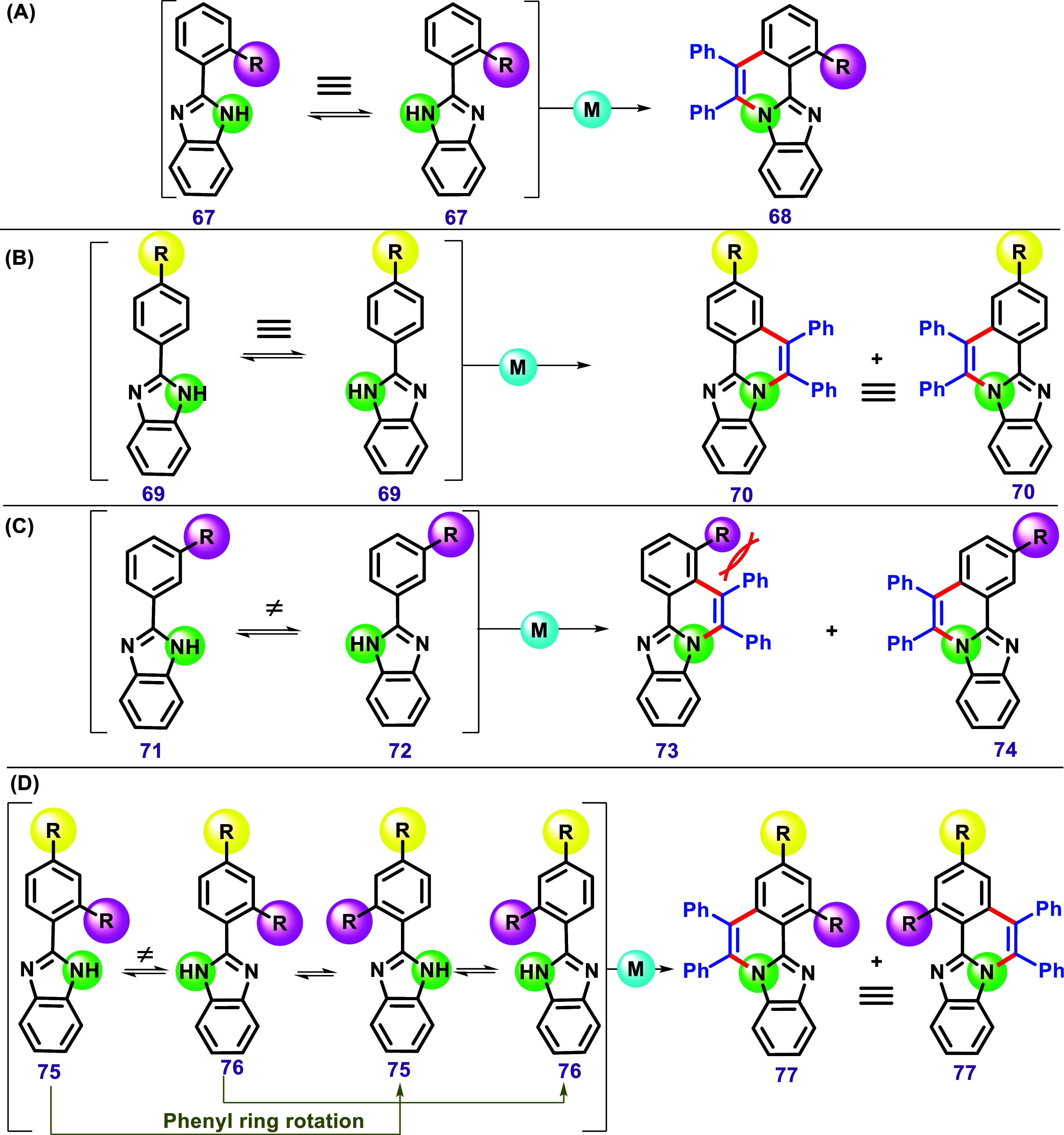
A Representation of the C–H/N–H Annulation of 2-Phenyl-1*H*-Benzo­[*d*]­Imidazole with Different Substitution Patterns on the Phenyl Ring: (A) Bearing an *Ortho*-Substituted Phenyl, and (B) a *para*-Substituted Phenyl, Resulting in the Formation of One Product in Both Cases. (C) A Similar Reaction with a *meta*-Substituted Phenyl, Yielding Two Isomers, and (D) a 2,4-Disubstituted Phenyl, Forming One Product.

A more intriguing case may arise from substrates bearing a substituent in the imidazole group coupled with a substituent in the *meta* position on the aryl group. As illustrated in Scheme 13, combining the ideas discussed previously (Schemes 11 and 12) could theoretically result in four products.

**Scheme 13:**
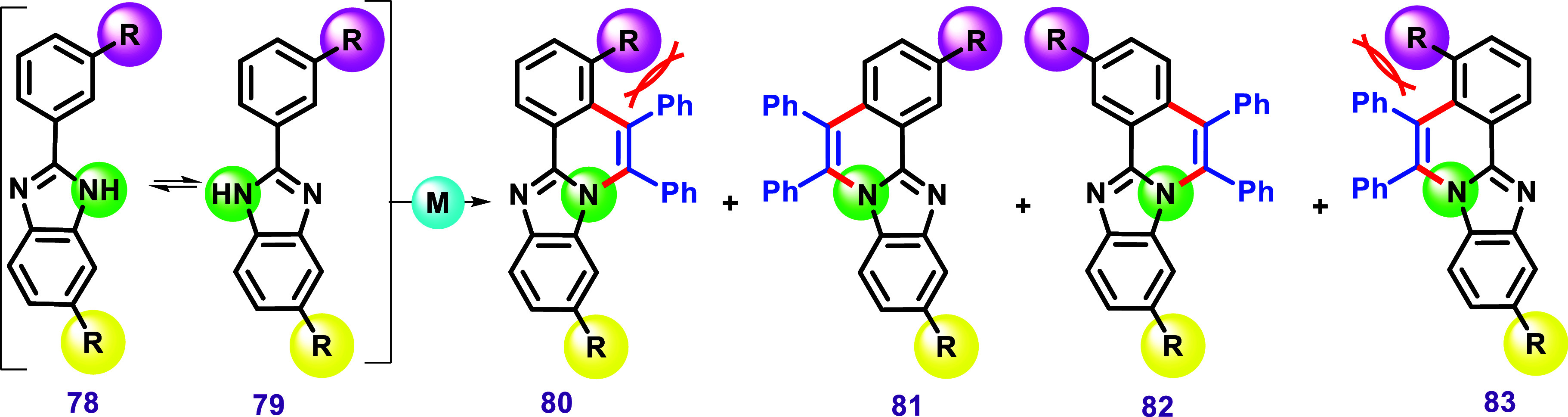
A Generic Representation of the C–H/N–H Annulation of 2-Phenyl-1*H*-Benzo­[*d*]­Imidazole Bearing a Substituent in the Benzimidazole Core and a *Meta*-Substituent Attached to the Phenyl Ring, with the Possible Formation of Four Isomers.

So far, the discussion of isomer formation has focused on the structural diversity of imidazole as a reaction substrate using diphenylacetylene. However, another essential aspect in studying such annulation reactions involves a nonsymmetrical alkyne. In these cases, where the alkyne contains two different groups, a mixture of isomers can be formed due to the different positions of the alkyne substituents (Scheme 14).

**Scheme 14:**
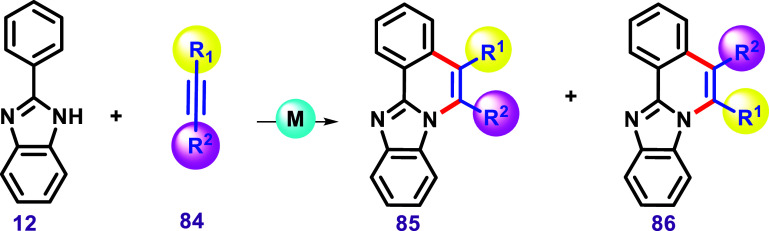
A Generic Representation of C–H/N–H Annulation of an Imidazole Using a Non-Symmetrical Disubstituted Alkyne Leads to the Formation of Two Possible Isomers.

Even more complex cases of isomerism involving different combinations of imidazoles with disubstituted alkynes are theoretically possible. For instance, when reacting with a nonsymmetrical alkyne, using a substrate containing a benzimidazole substituent in the core and a *meta*-substituent in the phenyl group could yield eight isomers. It is unsurprising that, to our knowledge, no such examples have been published to date.

However, it is crucial to highlight that electronic and/or steric factors can significantly influence the reaction pathway, leading to a preferred product or, at the very least, a specific product ratio. Depending on the substrate and/or the catalyst, a particular organometallic intermediate may not preferentially form. Another factor to consider is the potential repulsion between the substituent in the *meta* position and the inserted phenyl groups. Moreover, steric and electronic considerations in the case of nonsymmetrical alkynes can also favor the formation of a specific product. In the following examples, several cases involving issues of isomerism and selectivity will be discussed.

## 5 C–H/N–H Annulation of Imidazole Using Internal Alkynes

One of the earliest examples of C–H/N–H annulation reactions of imidazole was described in 2013 by Kavitha et al.^119^ The authors developed a protocol using [RuCl_2_(*p*-cymene)]_2_ as a catalyst and Cu­(OAc)_2_·H_2_O as an oxidant in toluene, affording the product **23** in 88% yield (Scheme 15). Various imidazoles, featuring electron-donating (EDG) and electron-withdrawing groups (EWG), were subjected to this protocol, affording products with good yields. These reactions were also carried out in PEG-400/H_2_O as the solvent, achieving products in similar yields compared to those obtained using toluene at room temperature. Additionally, the recyclability of the catalyst and PEG-400 was demonstrated, with minimal loss of activity after several cycles.

**Scheme 15:**
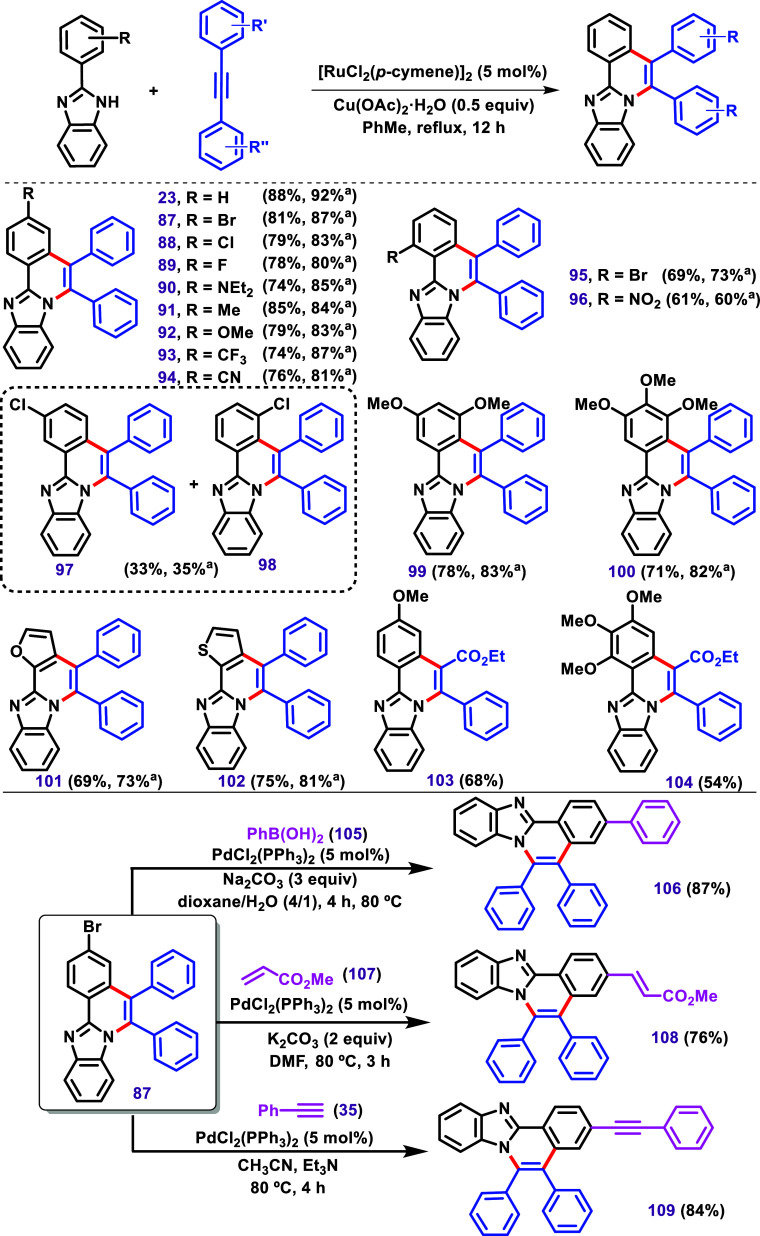
C–H/N–H Annulation between 2-Aryl Benzimidazoles and Alkynes via Ruthenium Catalysis Using Toluene or PEG-400, as Described by Kavitha et al. (ref 119)­^a^

a yield obtained using PEG-400/H_2_O (4:1) as solvent, 12 h at r.t.

The substrate bearing a *meta*-chlorine as a substituent resulted in a mixture of products (**97** and **98**), as previously described in Scheme 12C. The nonsymmetrical ethyl phenylpropionate was also used in two cases under the described conditions, yielding the respective products **103** and **104**. These products were characterized through NOE experiments as the major isomers, as reported by the authors. The well-established palladium-catalyzed coupling conditions of Suzuki, Heck, and Sonogashira reactions were then applied to compound **87**, forming more conjugated molecular systems **106**, 108, and **109**, respectively.

C–H activation protocols typically employ catalysts based on second–row transition metals, such as ruthenium and rhodium. While these catalysts are highly efficient and widely used, they can be expensive and sometimes sensitive to moisture and air, which limits their application. As a result, there is a continuous interest in discovering more stable and cost-effective catalysts for C–H activation.^120^ In this regard, Dutta and Sen, in 2018, developed a protocol for promoting C–H/N–H annulation reactions of imidazole using alkyne with the [Cp*CoI_2_]_2_ catalyst (Scheme 16).^121^ This protocol led to the formation of compounds when different imidazoles were subjected to the reaction.

**Scheme 16:**
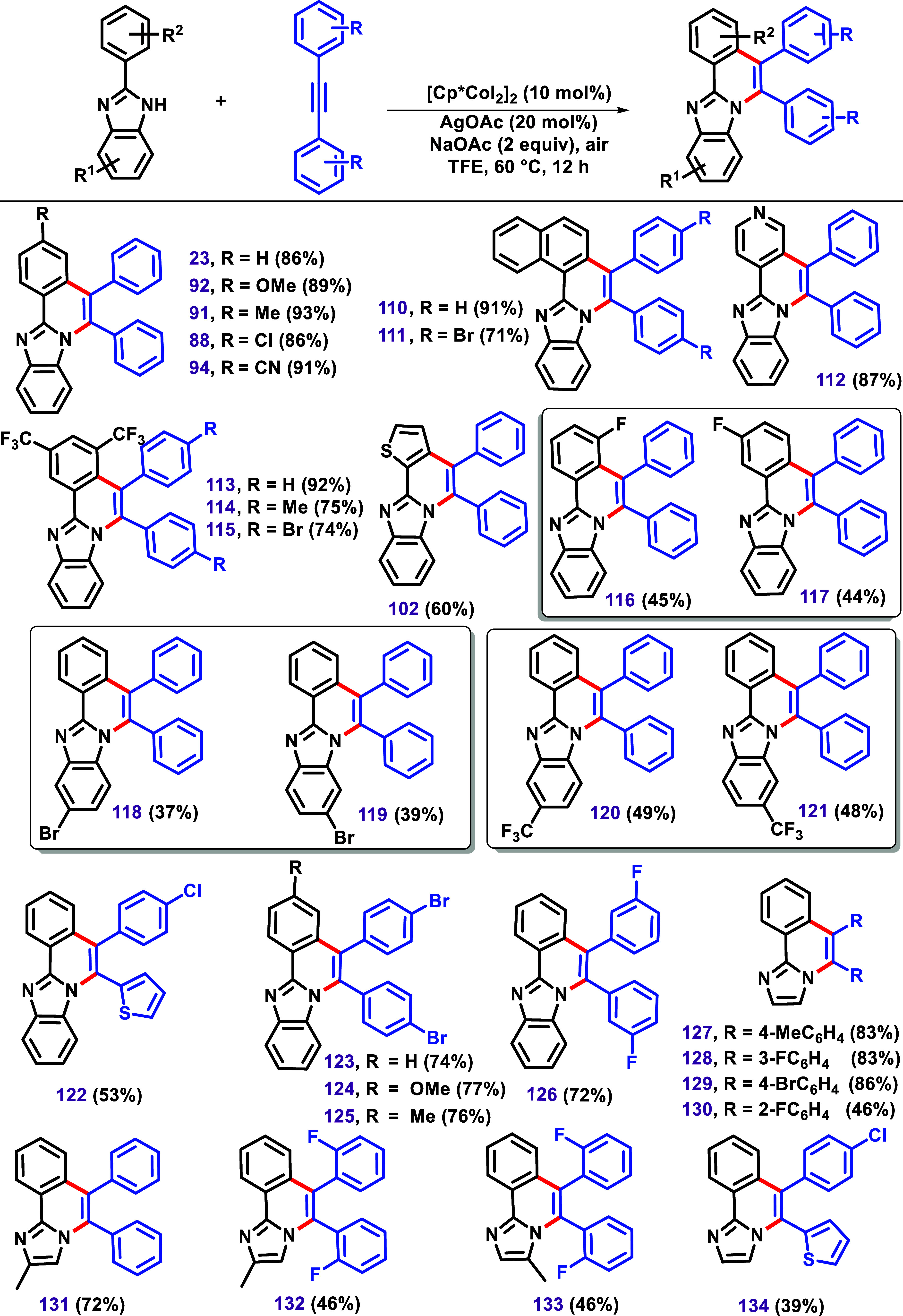
C–H/N–H Annulation between 2-Aryl Benzimidazoles and Alkynes via [Cp*CoI_2_]_2_, Described by Dutta and Sen.^121^

A substrate bearing a *meta*-fluoro substituent in the phenyl ring formed two isomers, **116** and **117**, in nearly equimolar proportion. This result is similar to the case of using *meta*-chlorine, as described by Kavitha et al.^119^ Furthermore, two examples of nonsymmetrical benzimidazoles bearing a bromide and trifluoromethyl groups in the benzimidazole moiety were used, leading to the almost equimolar formation of two regioisomers **118/119** and **120/121**. The products **120** and **121** were further confirmed by X-ray studies. These results align with the previous discussion on isomer formation (Scheme 11B). The authors also described products obtained by using nonsymmetrical alkynes. In this case, the product **122** was achieved as the major diastereomer.

Examples using 2-aryl imidazoles were also investigated by the authors, leading to the products **127**–**134** in 81–88% yield. The reaction between 4-methylimidazole and diphenylacetylene could result in two isomers; however, the authors reported only the formation of **131** in 72% yield. Interestingly, using bis­(2-fluorophenyl)­acetylene, an equimolar mixture of **132** and **133** was obtained. The nonsymmetrical alkyne 4-chlorophenyl-2-thiophenylacetylene yielded **134** in 39% yield, while the other possible isomer was not described. The authors reported that compound **110** displayed intense blue fluorescence emission centered at 407 nm.

Another example of the application of first-row metals in catalysis to carry out a C–H/N–H annulation was reported by Obata et al. in 2019 (Scheme 17).^122^ Ni­(OAc)_2_ and 4,4-di-*tert*-butyl-2,2-dipyridyl (dtbbpy, **135**) promoted C–H/N–H annulation of imidazole, producing **23** in 95% yield (Scheme 17A). The scope of this reaction was investigated using various aromatic and aliphatic alkynes. Interestingly, when phenylpropyne was used, product **139** was obtained regioselectively in 78% yield, with its structure confirmed by X-ray crystallographic analysis. Furthermore, the authors described some examples using the Ni­(cod)_2_/dtbbpy/no base (KO^***t***^Bu) system (Scheme 17B). These results indicated that Ni­(II) and Ni(0) can promote C–H/N–H annulation of imidazole.

**Scheme 17:**
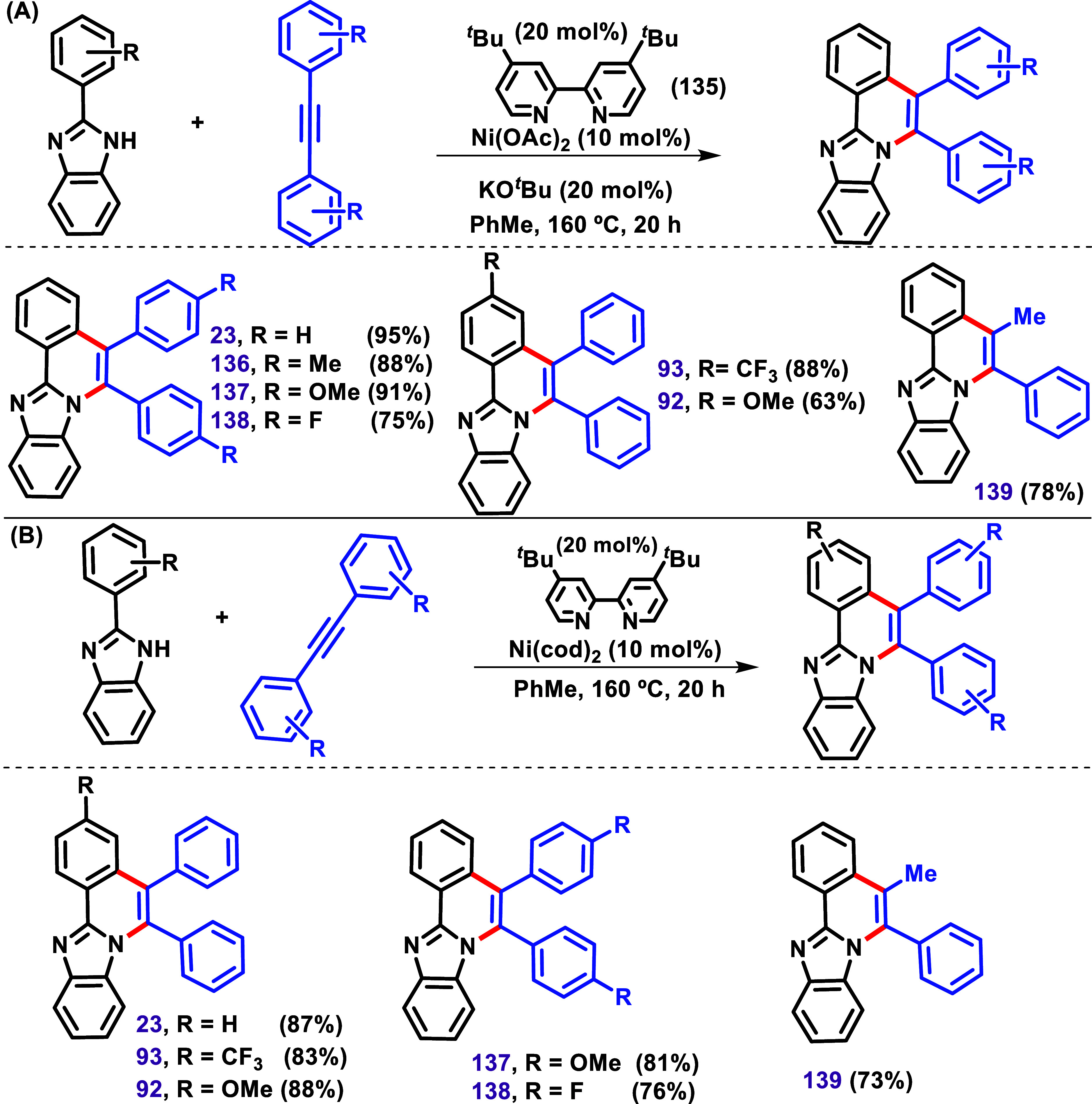
C–H/N–H Annulation between 2-Aryl Benzimidazoles and Alkynes (**A**) via Ni­(OAc)_2_ and 4,4-di-*tert*-Butyl-2,2-Dipyridyl (dtbbpy, **135**) and (**B**) via Ni­(cod)_2_/dtbbpy/No Base System Catalysis Described by Obata et al. In 2019.^122^

The previous examples described the mono-oxidative annulation of 2-aryl benzimidazoles with alkynes under Ru­(II), Co­(III), Ni­(II), and Ni(0) catalysis. However, in 2017, Villar et al. presented a different product pattern under [Cp*RhCl_2_]_2_ catalysis (Scheme 18A).^123^ The authors have described Rh­(III)-catalyzed double-oxidative annulation of 2-aryl benzimidazoles with alkynes to afford N-doped cationic polycyclic aromatic hydrocarbons (PAHs). In contrast, other metal complexes such as [Co­(III)], [Ir­(III)], [Pd­(II)], or [Ru­(II)] were inactive in this transformation. Different substrates were subjected to the optimized conditions to afford products with good yields. Using the nonsymmetrical alkynes 1-phenyl-1-propyne, product **148** was obtained regioselectively in 74% yield.

**Scheme 18:**
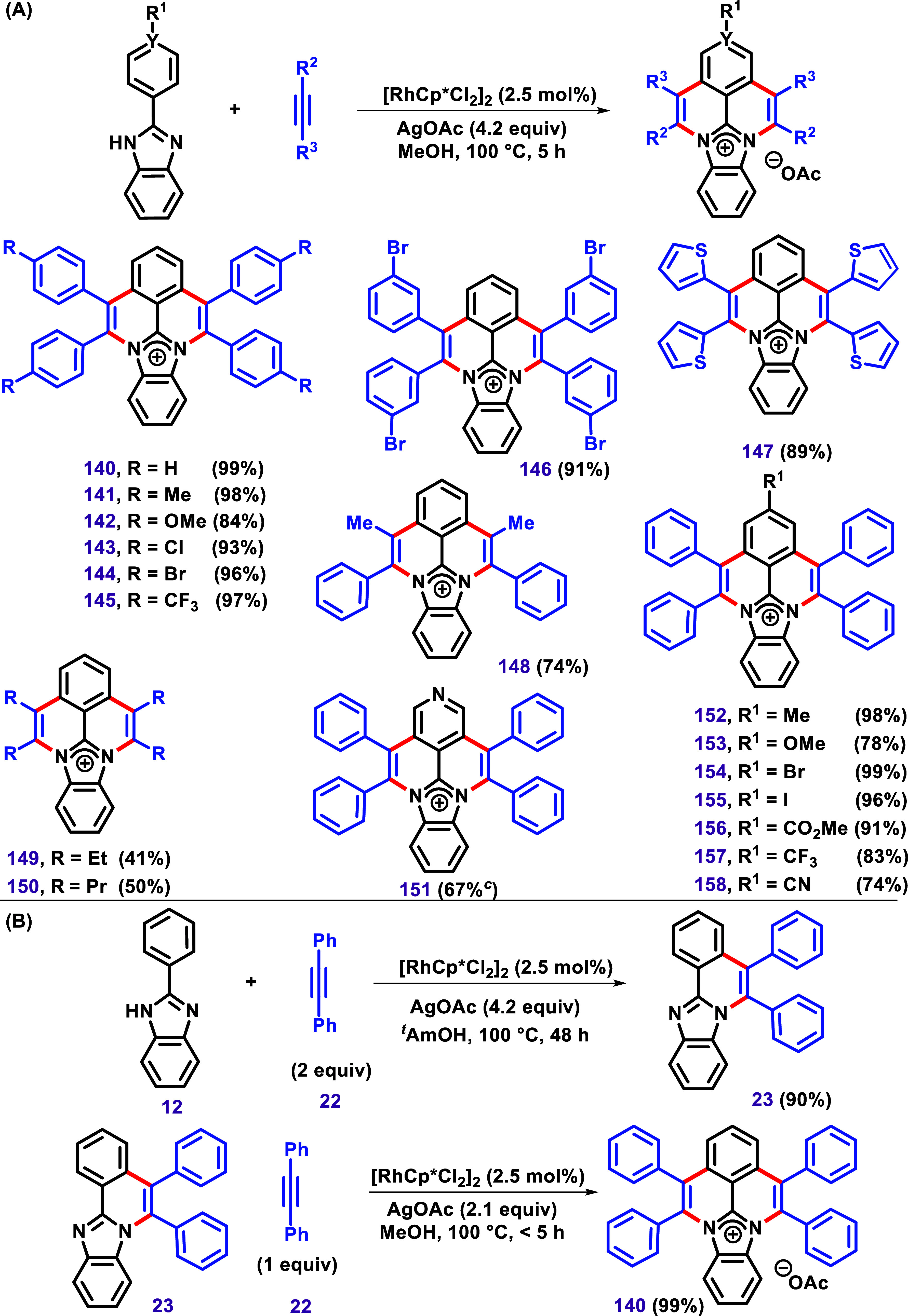
(A) Rh­(III)-Catalyzed Double C–H/N–H Activation and Annulation between 2-Aryl Benzimidazoles and Alkynes to Afford *N*-Doped Cationic Polycyclic Aromatic Hydrocarbons and (**B**) the Formation of the Mono-Cyclized Compound **23** under [Cp*RhCl_2_]_2_/AgOAc Catalyst System in *tert*-Amyl Alcohol, Followed by its Conversion to **140** as Described by Villar et al.^123^

The authors described the formation of the monocyclized compound **23** in 90% yield under [Cp*RhCl_2_]_2_/AgOAc catalyst system in *tert*-amyl alcohol. This product was then subjected to the optimized reaction conditions, and complete conversion to **140** occurred in less than 5 h, with the product being isolated in 99% yield. This result supports the formation of **23** as an intermediate (Scheme 18B).

Replacement of the acetate anion with PF_6_^–^, SbF_6_^–^, OTf ^–^, and BArF_4_^–^ afforded similarly stable compounds. The authors described that all compounds exhibited a pale yellow color with strong blue-light emission under irradiation at 254 nm, with absorption centered at 290 nm. The molar extinction coefficients strongly depended on the position of the *para*-substitution.

Charged organic molecules possessing π-conjugated systems, as described by Villar et al. (Scheme 18),^123^ are important in the development of organic electronic materials. Inspired by these compounds, Chen et al. synthesized the doped cationic PAHs **159**–**164** (Scheme 19) and investigated their optical properties.^124^ The compounds exhibited higher luminescence efficiency than the reported terminal-type organic salts and displayed aggregation-induced emission enhancement performance and tunable emission. The authors demonstrated that rationally dispersing the surface charge of the large π cation by regulating donor–acceptor distribution increases anion-π^+^ interactions and suppresses the free rotation of phenyl groups. This electronic phenomenon resulted in fluorescence enhancement in the solid state.

**Scheme 19:**
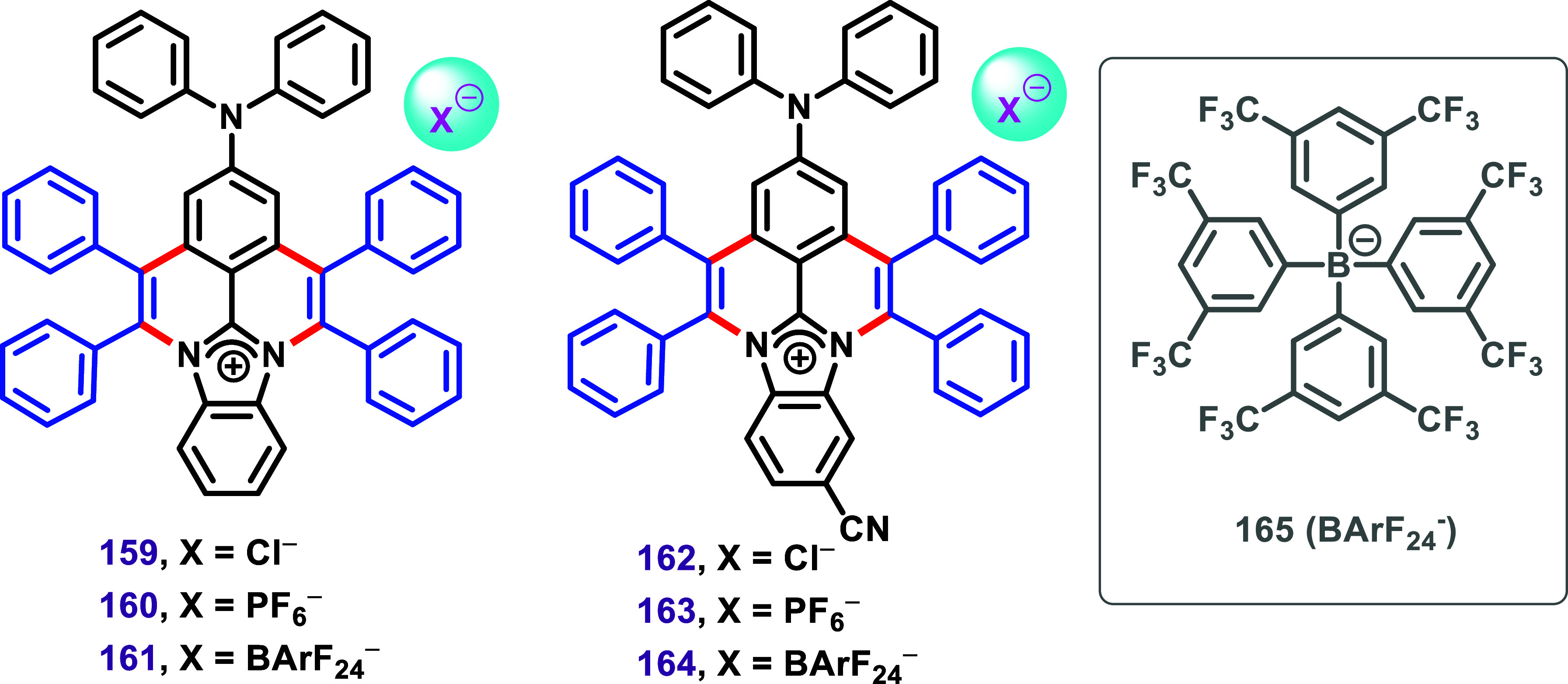
Polycyclic Aromatic Hydrocarbon (PAH) **159**–**164**, Synthesized by Chen et al., with High Solid-State Luminescence Efficiency Based on Anion-π^+^ Interactions Tuning Strategy.^124^

Furthermore, tuning the steric hindrance and charge dispersion of the counterions, using the bulky BArF_24_^–^ anion (**165**), enabled the preparation of sublimable, pure organic ionic materials with high solid-state luminescence efficiency. A vacuum-evaporated OLED was also demonstrated, based on a pure organic salt with bright yellow emission. According to the authors, these results may offer a new alternative material for organic optoelectronics applications.

The examples discussed have demonstrated how benzimidazole has been utilized as a substrate in annulation reactions through C–H bond activation. However, even more conjugated systems can be built by choosing the imidazole class. For instance, Zheng and Hua described π-extension of 2-aryl-phenanthroimidazoles via rhodium­(III)-catalyzed C–H/N–H annulation (Scheme 20).^125^ Various 2-aryl-phenanthroimidazoles were subjected to the optimized conditions, producing products **166**–**180** in good yield. However, substrates bearing EDG OMe and NMe_2_, as well as a fluorinated substrate, led to products **169**, **170**, and **171** in lower yields (Scheme 20A). Product **176** was obtained as the only regioisomer, confirmed by X-ray analysis. The authors also explored the possibility of reaching product **166** in a one-pot protocol using 9,10-phenanthraquinone (**181**) as a substrate (Scheme 20B). This reaction, catalyzed by [Cp*RhCl_2_]_2_, led to the formation of **166** in 56% yield. In contrast, the [RuCl_2_(*p*-cymene)]_2_ catalyst promoted the same reaction, affording the product in 32% yield.

**Scheme 20:**
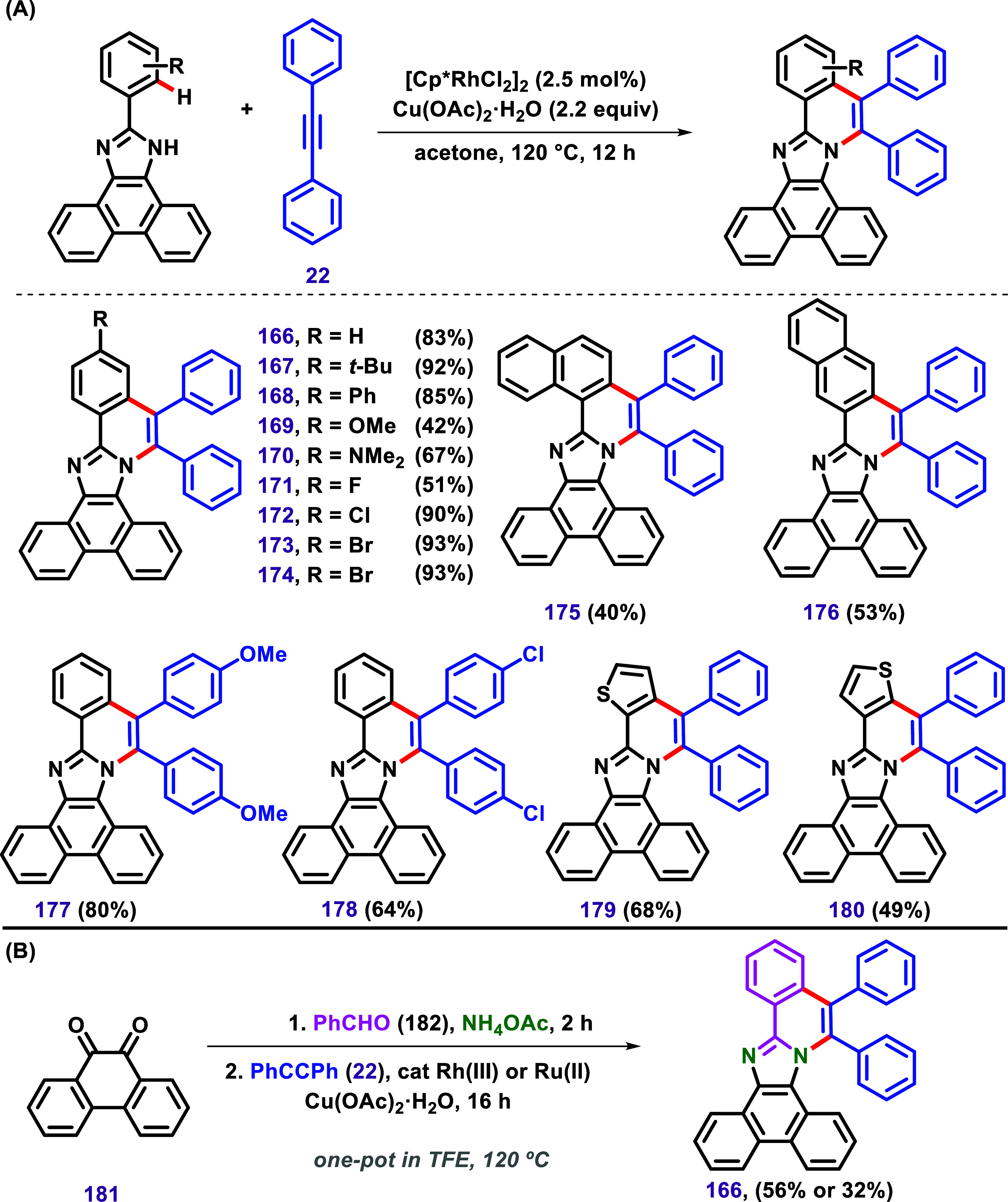
(A) C–H/N–H Annulation between Phenanthroimidazoles and Diphenylacetylenes via Rhodium Catalysis and (**B**) C–H/N–H Annulation between 9,10-Phenanthraquinone (**181**) and Diphenylacetylene (**22**) in a One-Pot Protocol via Rhodium or Ruthenium Catalysis, as Described by Zheng and Hua.^125^

Curiously, the nonsymmetrical alkyne **182** resulted in two products, **183** and **184**, both confirmed by X-ray diffraction, with the phenyl at different positions, suggesting a possible rearrangement (Scheme 21A). The use of this alkyne for C–H/N–H annulation was described by Obata et al. in 2019 using Ni­(OAc)_2_ (Scheme 17),^122^ and also by Yang et al. using [RuCl_2_(*p*-cymene)]_2_ (Scheme 23).^126^ In both cases, neither the formation of two isomers nor a rearrangement was described, which suggests that [Cp*RhCl_2_]_2_ may promote such transformation. To explain rearrangement, Zheng and Hua proposed the mechanism (Scheme 21B) via the β-elimination of rhodacycle intermediate **185**, resulting in **186**, followed by intramolecular allene insertion to afford **187**, and finally, reductive elimination leading to **184**.^125^

**Scheme 21:**
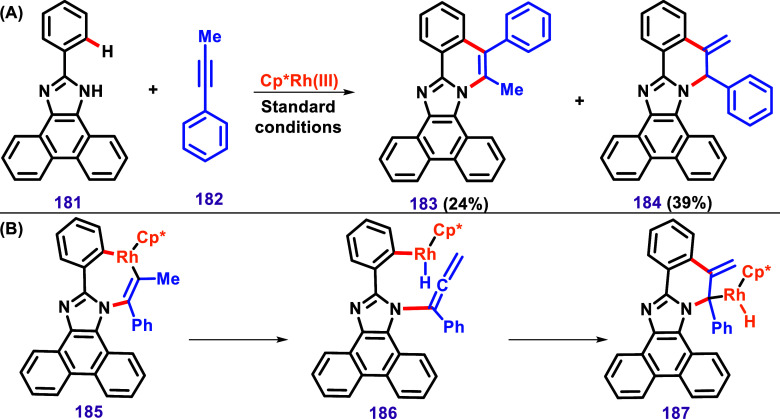
(A) Formation of Products **183** and **184** via a Rearrangement. (B) The Proposed Mechanism of This Transformation, as Described by Zheng and Hua.^125^

**Scheme 22:**
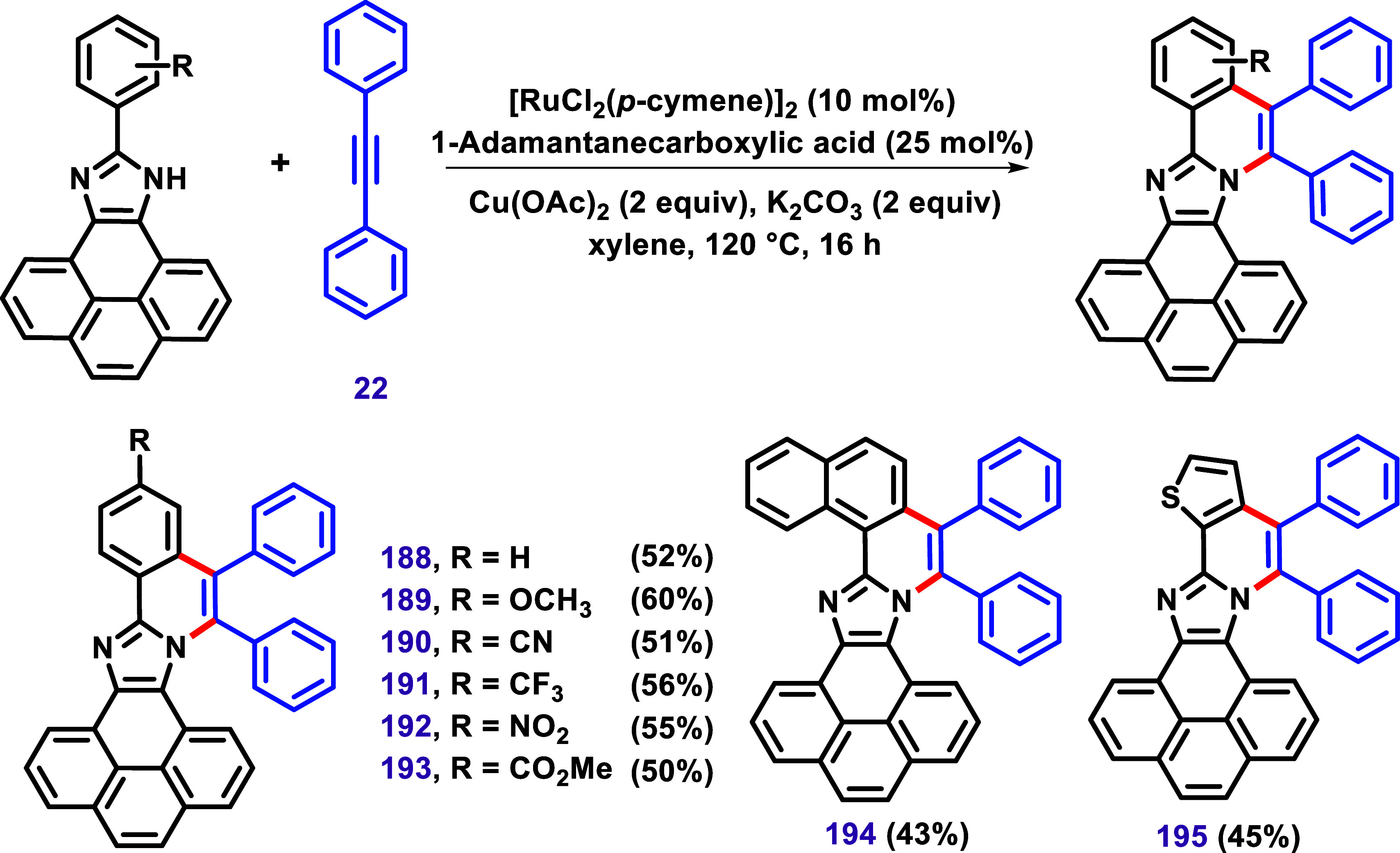
C–H/N–H Annulation of 10-Aryl-Pyrenoimidazoles Using [RuCl_2_(*p*-cymene)]_2_/Cu­(OAc)_2_ as a Catalyst System Described by Karthik et al.^130^

**Scheme 23:**
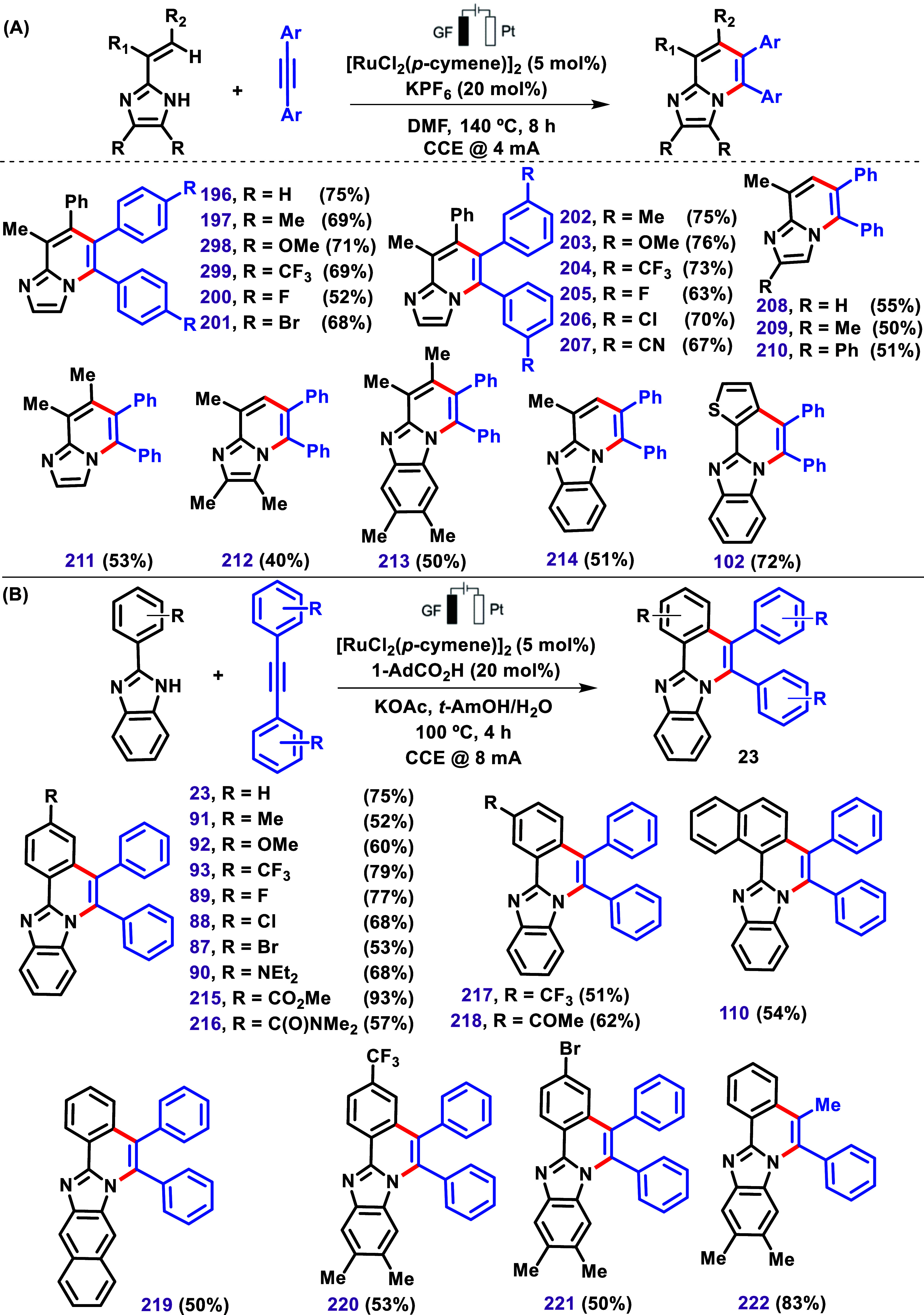
C–H/N–H Annulation under Ruthenium-Catalyzed Electrochemical Dehydrogenative Reactions between Alkynes and (**A**) 2-Vinyl-Imidazole/2-aryl-Benzimidazole and (**B**) 2-Aryl-Benzimidazole, as Described by Yang et al.^126^

The product **179** was investigated as a potential chemosensor for the detection of cations. Photophysical studies revealed that adding Fe^3+^ to the **179** solution resulted in a 4.6-fold enhancement and a slight blue shift in emission. This modification was also visible to the naked eye under UV light. The authors proposed that this optical response is due to the interaction of the bidentate ligand moiety of **179** with Fe^3+^. In this context, the fused ring could increase structural rigidity and alter the π-electron distribution, leading to modifications in the optical properties of **179**. Adding other cations such as Li^+^, Ag^+^, Zn^2+^, Cu^2+^, Ni^2+^, and Co^2+^ did not significantly alter the optical response, whereas Cr^3+^ caused some interference.

1*H*-Phenanthro­[9,10-*d*]­imidazole is a more extensively conjugated π-system than 1*H*-benzimidazole. However, this class of compounds could be further conjugated by incorporating molecular systems such as 10-phenyl-9*H*-pyreno­[4,5-*d*]­imidazole (Scheme 1). Due to its optical properties, the pyrene core, a well-studied molecule, has attracted significant scientific interest.^127–129^ This growing interest in such compounds inspired Karthik et al. to develop a C–H/N–H annulation of 10-arylpyrenylimidazole using [RuCl_2_(*p*-cymene)]_2_/Cu­(OAc)_2_ as a catalyst system (Scheme 22).^130^ The synthetic protocol was successfully applied to various substrates bearing EDG and EWG, leading to compounds **188**–**195** in yields ranging from 43% to 60%.

The authors presented systematic studies of the optical properties of this molecular core through UV–vis and fluorescence spectroscopic investigations. They observed that while vibronic features in the UV–vis spectra remained consistent for pyrenylimidazole, the relative intensities and symmetry of the absorption peaks varied significantly with the different substituents. Additionally, the absence of a mirror image relationship between the absorption and fluorescence spectra, commonly seen in PAHs systems such as pyrene, along with a large Stokes shift (ca. 3750 cm^–1^), led the authors to conclude that there is a significant structural difference between the ground and excited states of the products. Compounds **194** and **195** exhibited further red-shifting of the absorption spectrum compared to the simpler example, **188**. This result was attributed to the extended π-conjugation in **194** and **195**.

The Stokes shift (the difference between λ_em_ and λ_abs_) observed for the products ranged from 2300 to 7000 cm^–1^. The authors found this range striking, attributing it to the polarization of the electron density between the pyrene and imidazole subunits. The large Stokes shift suggests a reduced overlap between the absorption and emission spectra, with compound **192** exhibiting negligible overlap. The absolute fluorescence quantum yields in chloroform were also measured and found to be sensitive to the substituents, with values ranging from 0.09 (**192**) to 0.65 (**193**). Fluorescence lifetimes were determined using the time-correlated single photon counting (TCSPC) technique, resulting in χ^2^ values ranging from 1.2 ± 0.1 for the compounds. The optical properties of the products were evaluated in different solvents, and results showed that solvent polarity did not significantly interfere in UV–visible absorption. However, the emission spectrum was red-shifted with increasing solvent polarity, depending on the nature of the substituent. DFT calculations, electrochemical properties, and thermal properties were also investigated.

Metal-catalyzed C–H activation reactions often require superstoichiometric amounts of chemical oxidants, particularly copper- or silver-based metal oxidants. More recently, combining organometallic C–H activation with electrocatalysis has emerged as a promising alternative to replace metal-based chemical oxidants.^131–133^ In this context, in 2020, Yang et al. reported a ruthenium-catalyzed electrochemical dehydrogenative annulation of imidazoles with alkynes using an undivided cell setup equipped with a graphite felt (GF) anode and a platinum cathode (Scheme 23).^126^ Various combinations of imidazole and alkynes were subjected to this protocol, resulting in products with 40–76% yields (Scheme 23A). After further optimization of the reaction conditions, 2-aryl benzimidazole was also subjected to C–H/N–H annulation, affording products in yields ranging from 50% to 93% (Scheme 23B). Interestingly, the use of nonsymmetrical 1-phenyl-1-propyne afforded the product **225** with a high degree of regioselectivity, as reported by the authors.

In 2021, Kumar et al. described an electricity-induced microflow C–H/N–H alkyne annulation with 2-aryl benzimidazole, performed without the use of an expensive oxidant (Scheme 24).^134^ The optimization of the reaction was carried out using a novel microelectro flow heating reactor (μ-EFR), which employed electricity as a green oxidant to drive the process and utilized [RuCl_2_(cymene)]_2_ as the catalyst. Various imidazoles were tested to evaluate the reaction’s efficiency, demonstrating high tolerance for both imidazoles and alkynes.

**Scheme 24:**
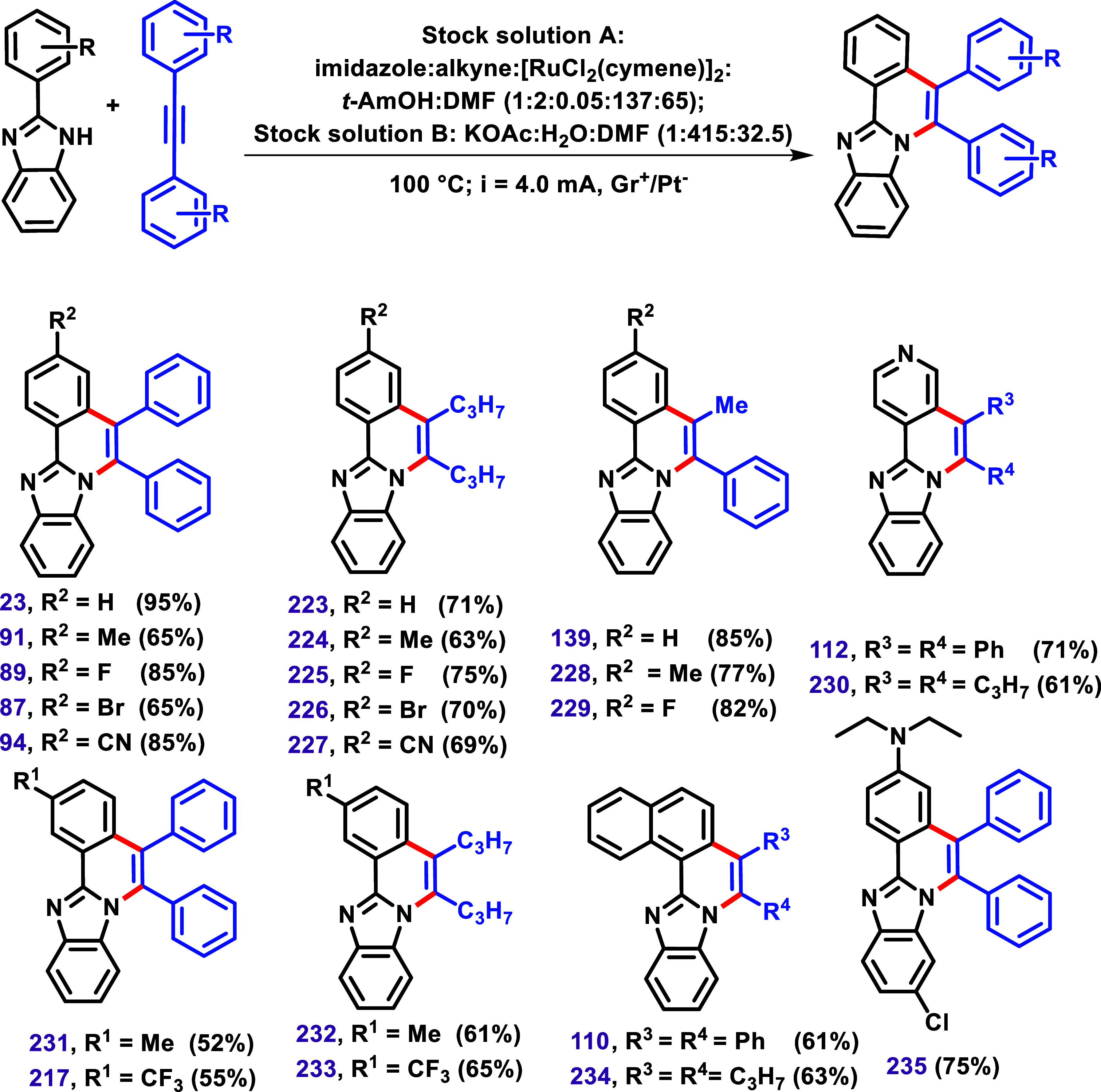
Electricity-Induced Micro-Flow C–H/N–H Alkyne Annulation with 2-Aryl Benzimidazole without the Use of Expensive Oxidant Described by Kumar et al.^134^ Reaction Conditions: Stock Solution **A**: imidazoles:alkynes:[RuCl_2_(cymene)]_2_:*t*-AmOH:DMF (1:2:0.050:137:65); Stock Solution **B**: KOAc:H_2_O:DMF (1:415:32.5), at 100 °C; Constant Current at 4.0 mA, Gr^+^/Pt^–^.

The authors reported that the reaction was site-selective, particularly in substrates with a *meta*-substituent (**217**, **231**–**233**), attributed to the steric hindrance. Additionally, they highlighted intriguing examples involving nonsymmetrical alkynes, which yielded site-selective annulated products **139**, **228**, and **229** with the alkyl group positioned distal and the π-motif proximal to the imidazole nitrogen. According to the authors, this outcome was attributed to attractive π–π dispersion interactions that govern the key migratory insertion step.

Subsequently, the same research group described a microphoto-flow reactor system designed to promote a similar chemical reaction (Scheme 25).^135^ This protocol developed a visible-light-driven modular microflow reactor, equipped with an integrated postsynthetic workup, to promote the C–H/N–H annulation between imidazole and alkynes. The reaction was optimized by varying solvents, base, light, catalyst, and temperature. The optimal conditions were identified as a combination of CHCl_3_/MeOH solvent, KOAc, [RuCl_2_(*p*-cymene)]_2_ catalyst at 30 °C.

**Scheme 25:**
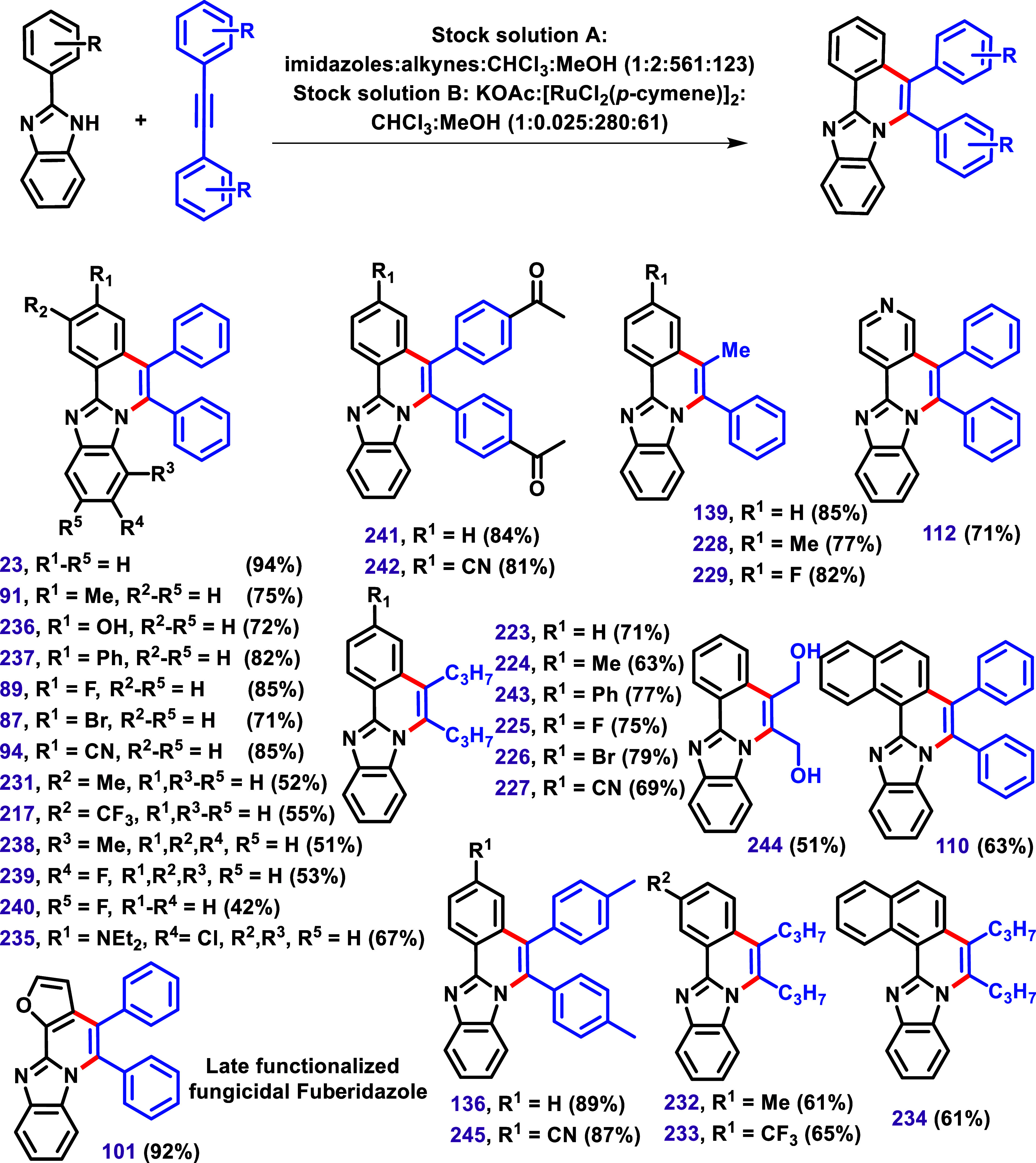
An Integrated Continuous Flow Visible Light-induced C–H/N–H Annulation between Imidazoles and Alkynes Described by Kumar and Singh^135^ Reaction Conditions: Stock Solution **A**: imidazole:alkynes:CHCl_3_:MeOH in a Molar Ratio (1:2:561:123); Stock Solution **B**: KOAc:[RuCl_2_(*p*-cymene)]_2_:CHCl_3_:MeOH in a Molar Ratio (1:0.025:280:61).

These optimized conditions were subsequently applied to various 2-aryl imidazoles bearing either EWG or EDG, as well as to diarylacetylenes, yielding products in yields ranging from 42% to 94%. Similar to the previous example (Scheme 24),^134^ the authors reported that the photocatalyzed annulation reaction was found to be site-selective (**217**, **231**, **232**, and **233**) in cases involving a *meta*-substituent on the aryl imidazole, attributing this selectivity to steric hindrance. Furthermore, nonsymmetrical alkynes also produced site-selective annulated products **139**, **228**, and **229**.

Kawakami, Nohira, and Chatani reported an interesting case of nickel-catalyzed C–F/N–H annulation between benzimidazole bearing an *ortho*-fluorine substituent and alkynes via relatively underexplored C–F bond activation (Scheme 26A).^136^ Optimization studies identified the use of Ni­(cod)_2_ (10 mol %), Me_4_phen (**246**-5 mol %), and KO^*t*^Bu (1 equiv) in DMF at 100 °C as the optimal condition for transformation.

**Scheme 26:**
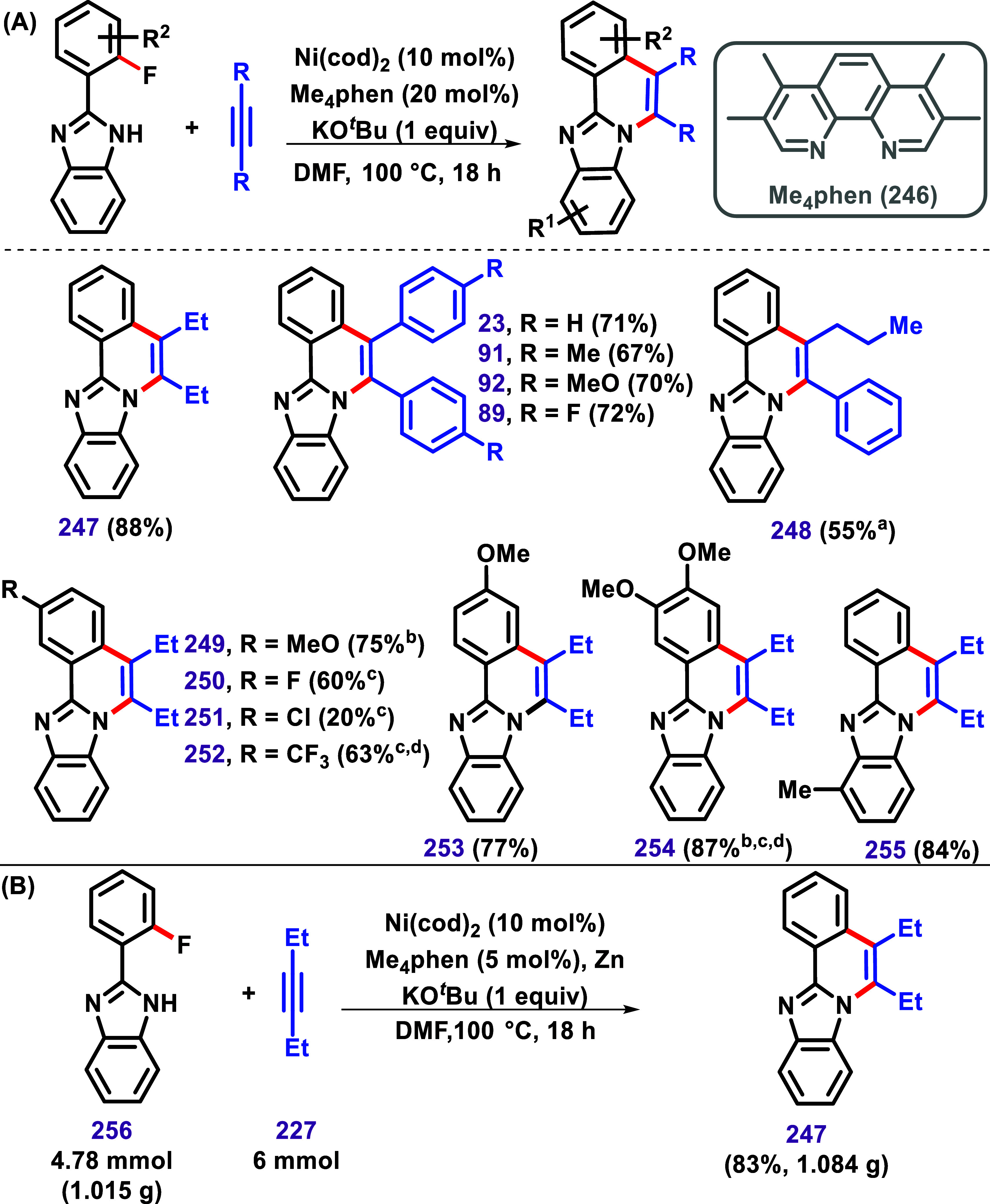
(A) Nickel-Catalyzed C–F/N–H Annulation between 2-(2-Fluorophenyl)-1*H* benzo­[*d*]­Imidazoles and Alkynes and (B) A benchtop-stable Ni­(OAc)_2_/Zn system applied in a gram-scale reaction to afford the product **247**Described by Kawakami, Nohira, and Chatani (ref 136)­^a^–^d^

a At 120 °C.
b LiOBut was used as a base.
c at 120 °C.
d Bpy (2,2′-dipyridine) was used instead of Me_4_Phen.

Subsequently, this protocol was applied to 2-(2-fluorophenyl)-1*H* benzo­[*d*]­imidazoles with various alkynes, resulting in compounds with yields ranging from 20% to 88%. Only one isomer was observed for product **248**, confirmed through X-ray crystallography; however, the authors did not discuss the possibility of isomer formation for other products. Furthermore, using an inexpensive and bench-stable Ni­(OAc)_2_/Zn proved effective, delivering product **247** in 83% yield, demonstrating the scalability of this method to gram-scale synthesis (Scheme 26B).

The examples discussed so far illustrate the C–H/N–H annulation using imidazole as a substrate to produce benzo­[4,5]­imidazo­[2,1-*a*]­isoquinolines. However, Meng et al. described the synthesis of this heterocycle via rhodium­(III)-catalyzed oxidative annulation of amidines with alkynes through sequential C–H bond activation (Scheme 27).^137^ In this new protocol, a double C–H activation was achieved in a one-pot fashion with the assistance of C=N and C–N bonds.

**Scheme 27:**
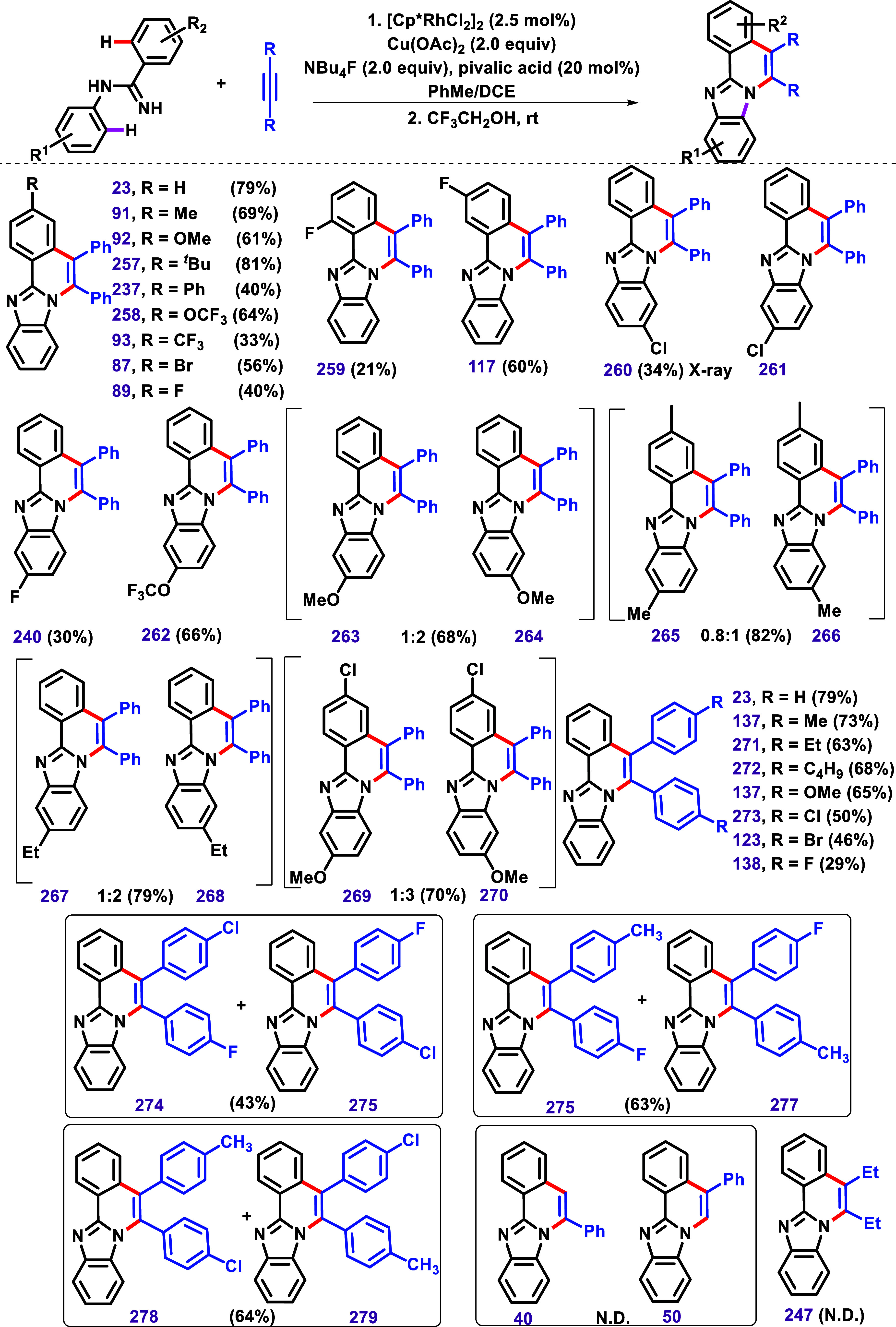
Rhodium­(III)-Catalyzed Oxidative Annulation of Amidines with Alkynes through Sequential C–H Bond Activation, Described by Meng et al.^137^

Upon optimizing the reactions, the authors applied the new protocol to diverse reagents, varying amidines, and alkynes. Various amidines, irrespective of their substituent and electronic properties, were successfully utilized in the reaction. Substrates bearing a *meta*-moiety (for instance, *meta*-F) on the *C*-phenyl ring led to the selective production of product **117**, associated with C–H bond activation with less steric hindrance. Substrates with EDG in the *N*-phenyl ring resulted in two regioisomers in higher yields.

Electron-neutral groups (ENG), EDG, and EWG on the alkyne were generally well tolerated, resulting in compounds with reasonable yields. However, the *ortho*-methyl of the phenyl ring failed to yield the product, which was attributed to steric effects. The cascade reactions of nonsymmetrical disubstituted alkynes produced two possible regioisomers in moderate yields. However, terminal alkynes did not deliver the desired product.

Yu et al.^138^ synthesized compounds **281** and **283** in a one-pot manner, starting with the preparation of 2-aryl-phenanthroimidazoles through a multicomponent reaction of 9,10-phenanthraquinone, an aryl aldehyde, and ammonium acetate in the presence of trifluoroethanol (Scheme 28). Next, 1,2-diphenylacetylene was directly added to the reaction mixture, using [Cp*RuCl_2_]_2_ as the catalyst and Cu­(OAc)_2_·H_2_O as oxidant, resulting in the formation of products **281** and **283**, which were characterized by single crystal X-ray diffraction. This protocol was based on the work of Zheng and Hua (Scheme 20A).^125^

**Scheme 28:**
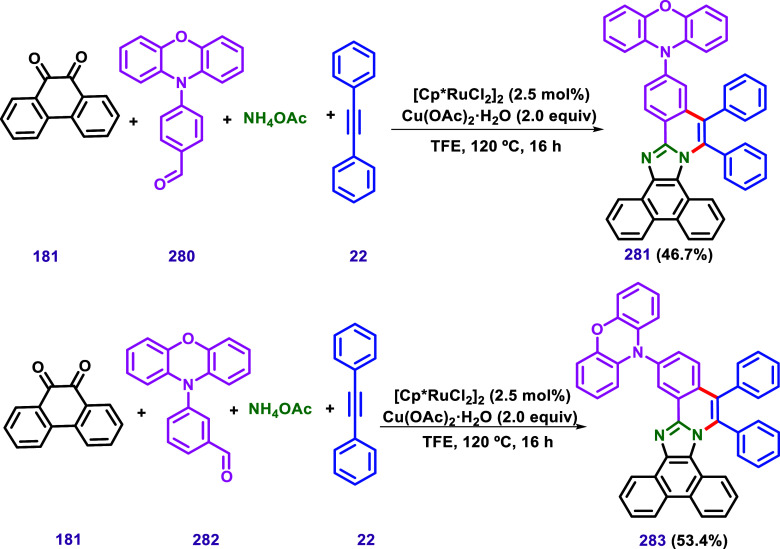
Phenanthro­[9′,10′:4,5]­Imidazo­[2,1-*a*]­Isoquinoline Derivatives **281** and **283** Containing a Phenoxazine Moiety Described by Yu et al.^138^

The absorption and fluorescence emission of both compounds in dichloromethane were investigated. The absorption spectra of the solutions of compounds **281** and **283** were strikingly similar, displaying two intense bands at 238 and 308 nm and one weak absorption band at 368 nm. Based on these results, the authors concluded that the positions of phenoxazine substituents do not affect the absorption spectra of either compound.

Compounds **281** and **283** exhibited strong fluorescence emission at 513 and 526 nm, respectively, with no vibronic features. The absence of a mirror-image relationship between the absorption and fluorescence spectra, along with the large Stokes shifts of 7681 cm^–1^ and 8162 cm^–1^ for compounds **281** and **283**, respectively, led the authors to conclude that there is a significant structural difference between the ground and excited states of the compounds.

The absolute fluorescence quantum yields (Φ_f_) in dichloromethane solutions were measured, resulting in 1.2% and 3.7% photoluminescence quantum yields for compounds **281** and **283**, respectively. The photoluminescence decay lifetimes were measured to be 5.91 and 6.47 ns using a TCSPC spectrometer.

The examples discussed so far illustrate variations in substrate scope, ranging from imidazole to more extended π-systems such as benzimidazole, phenanthroimidazole, anthraimidazole, and pyreneimidazole, and employ a variety of catalysts based on rhodium, ruthenium, cobalt, and palladium. In addition, some reports incorporate electrochemical, photochemical, and flow-based systems. Table 1 summarizes these reactions, highlighting the reaction conditions and protocols employed.

**Table 1:**
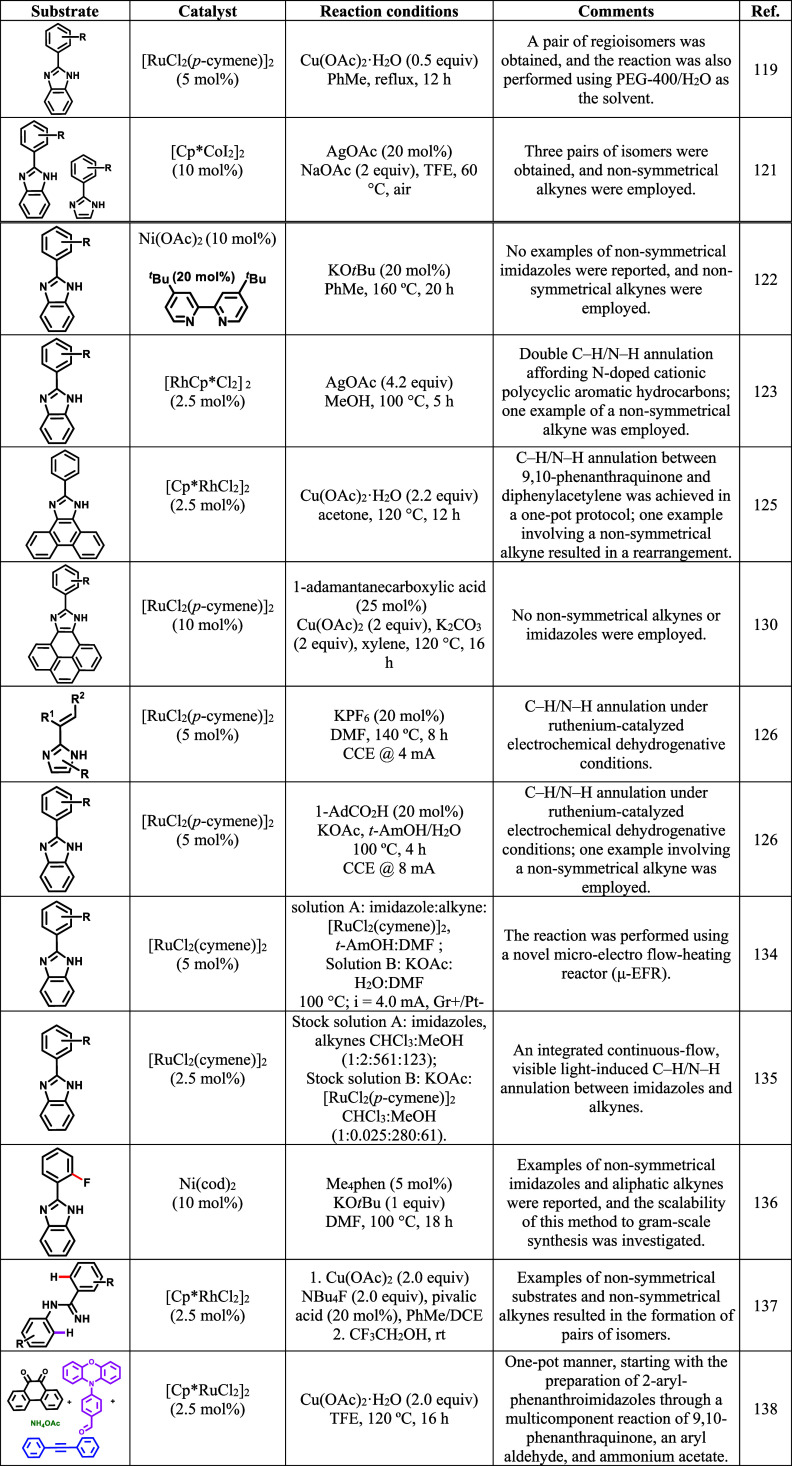
Summary of Transition-Metal-Catalyzed C–H/N–H Annulation of Imidazole Derivatives with Alkynes.

## 6 Lapimidazol as a Nonsymmetrical Substrate for C–H/N–H Annulation

As described in the previous examples, using symmetrical imidazole as substrates in C–H/N–H annulation appears preferable and is likely to prevent the formation of isomers. For this reason, few studies involving nonsymmetrical imidazoles have been carried out so far. However, in 2021, we reported a case in which a nonsymmetrical imidazole from β-lapachone (**286**), here named lapimidazole, was used to promote a C–H/N–H annulation.^139^

β-Lapachone (**286**) can be synthesized in a one-pot reaction using lawsone (**284**) as substrate^140^ or through the acid cyclization of lapachol (**285**) (Scheme 29A).^141^ The 1,2-dicarbonyl moiety of **286** can then be converted into an imidazole heterocycle by reacting with an aldehyde in the presence of ammonium acetate, forming aryl-lapimidazole. This class of compounds, particularly **288**, **289**, and **290** (Scheme 29B), has been extensively investigated for Pinto’s^142–144^ and Menna-Barreto’s groups^145–149^ as potential drug candidates against *T. cruzi* and *Mycobacterium tuberculosis*,^150^ as reviewed by Dias et al.^151^ In addition to the biological activity of these compounds, the fluorescence properties of lapimidazole and lapoxazole have also been studied as potential profluorophores for imaging NQO1 activity in tumor tissues.^152^ and as fluorescent stains for biological cells.^153–156^ For instance, compound **291** selectively stains mitochondria.^156^

**Scheme 29:**
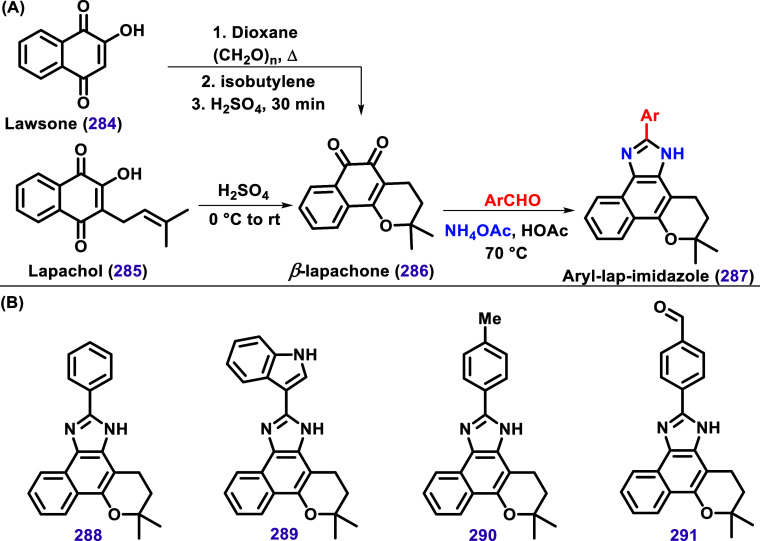
(**A**) Synthesis of β-Lapachone (**286**) from Lawsone (**284**) or Lapachol (**285**) and its Sequential Conversion to Aryl-Lapimidazole. (**B**) Examples of Aryl-Lapimidazole Derivatives **288**, **289**, and **290** Exhibiting Potent Biological Activity Against *T. cruzi* and Compound **291**, Capable of Selectively Staining Mitochondria.

The proton equilibrium of imidazole in lapimidazole results in the formation of two distinct structures due to the compound’s lack of symmetry concerning the aliphatic ring (Scheme 30). This equilibrium was investigated by Carvalho et al. using phenyl-lapimidazole (**288**).^157^ The ^1^H NMR spectra of **288** exhibited two signals associated with the N–H group, at 12.71 ppm (65%) and 13.20 ppm (35%), which were attributed to two interconvertible isomers, **288** and **292** (Scheme 30). ^1^H NMR experiments conducted in CDCl_3_ or DMSO-*d*_6_ with water showed no signal for N–H. The authors attributed this result to the fast exchange of the N–H proton with hydrogen chloride (in CDCl_3_) and water (in DMSO) present in these solvents. Irradiation at the signal at 12.71 ppm increased the signal’s intensity at 3.1 ppm (CH_2_ from the aliphatic ring) via Nuclear Overhauser Enhancement (NOE). Therefore, the authors attributed the signal at 13.20 ppm to isomer **292** and the signal at 12.71 ppm to isomer **288**. Experiments with a concentrated solution (50 mg/0.5 mL) in anhydrous DMSO-*d*_6_ revealed the coalescence of signals, indicating an intermolecular proton transfer process. The approximate 1:2 ratio of N–H signals (35% at 13.20 ppm and 65% at 12.71 ppm) under these conditions led the authors to conclude that isomer **288** is more stable than isomer **292**, which was supported by theoretical calculations and photophysical experiments.

**Scheme 30:**
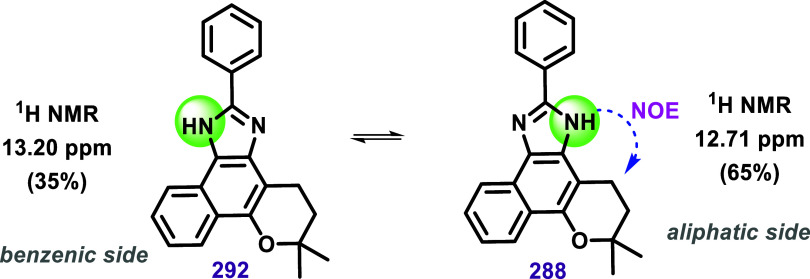
N–H Equilibrium of Lapimidazoles Isomers **288** and **292**, as Studied by Carvalho et al.^157^

In 2021, using this particular substrate, a nonsymmetrical imidazole, we developed a protocol for C–H/N–H alkyne annulation that incorporates a variety of functional groups, with substituents in *ortho*, *meta*, and *para* positions (Scheme 31).^139^ In this approach, [RhCp*Cl_2_]_2_/AgOAc catalytic system promoted C–H/N–H alkyne annulation of lapimidazoles bearing different substituents, forming two isomeric products. In all cases, the main product resulted from annulation on the benzene ring side of lapimidazole, whereas annulation on the aliphatic side yielded lower amounts, leading to products with good regioselectivity.

**Scheme 31:**
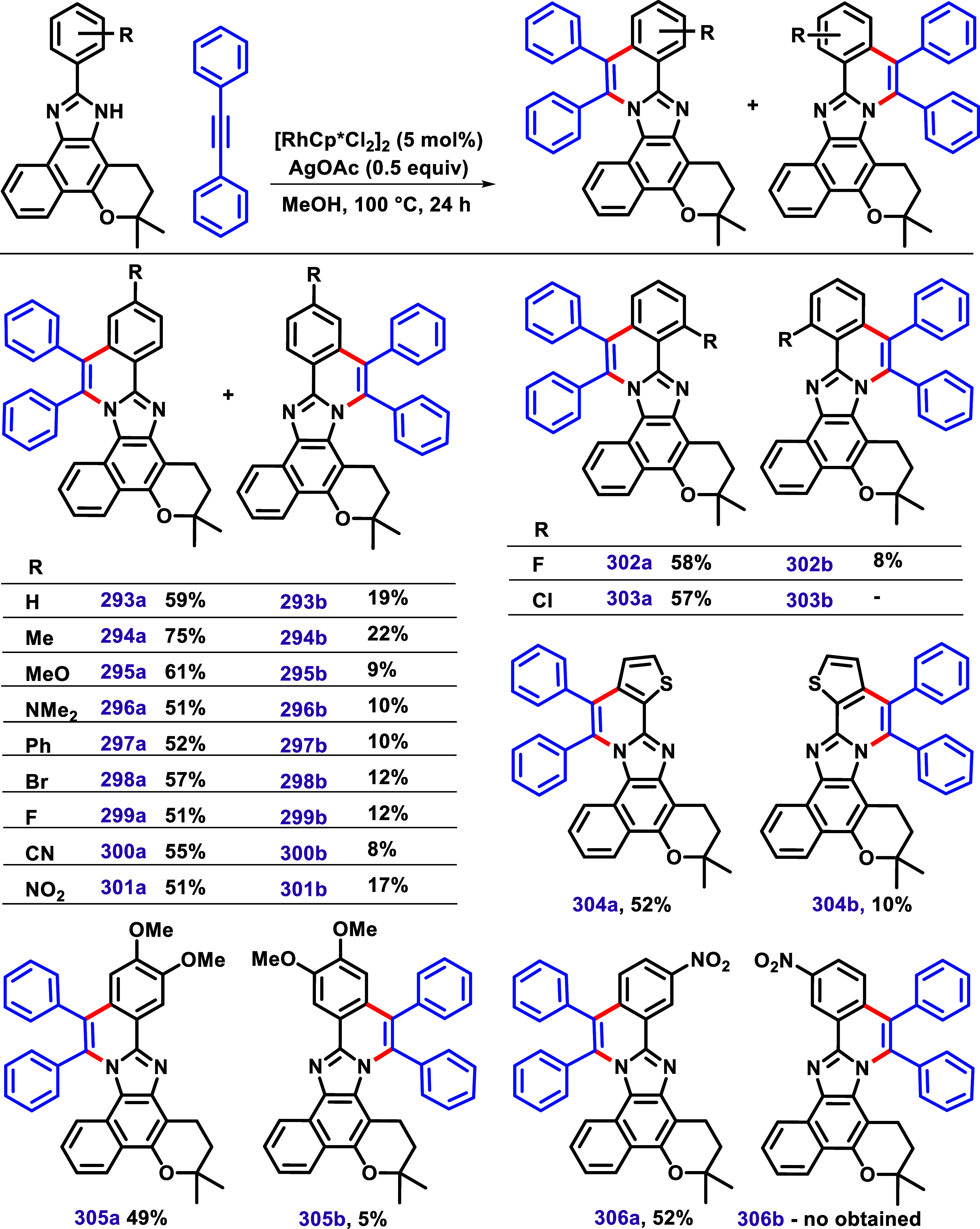
C–H/N–H Annulation of Aryl-Lapimidazole Using the [RhCp*Cl_2_]_2_/AgOAc Catalytic System Described by Dias et al.^139^

EDG and EWG in the *para* position were well tolerated in this protocol, resulting in products with yields of 51–75%. *Ortho*-substituents, such as chlorine and fluorine, also resulted in products with good yields. In the case of *ortho*-chlorine, only **303a** was achieved. The product **304a**, bearing thiophene, was obtained in 52% yield, with its isomer obtained in only 10%. The authors also described two examples bearing substituents in the *meta* position. Compounds **305a** and **305b** were synthesized similarly, and no other isomer was described.

Additionally, both compounds **305a** and **305b** were confirmed by X-ray diffraction studies. In the case of the *meta*-NO_2_ substituent, only the product **306a** was obtained. No products were achieved using lapimidazole-bearing indole and pyrimidine groups. The protocol developed was scaled up to the gram scale, yielding product **293a** in 53% yield, demonstrating the viability of performing this reaction on a large scale.

Studies using examples of nonsymmetrical imidazoles have generally shown the formation of equimolar isomers. In most of these cases, the authors employed substituents located relatively far from the reactive region of the molecule. Therefore, the electronic and/or steric effects may not be sufficient to induce selectivity. In the case of using lap-imidazoles, we observed a predominance of one isomer over the other, which may be attributed to the influence of the aliphatic ring on the reaction pathway.

To shed light on the mechanism and provide a possible explanation for the regioselectivity of the reaction, DFT studies were carried out. In these studies, the authors have described that coordination of the alkyne and its subsequent migratory insertion are less favorable in the pathway involving metalation of the nitrogen at the aliphatic site, resulting in a preference for annulation at the benzene side.

The authors selected the products **295a** and **295b**, bearing OMe as the EDG; **301a** and **301b**, bearing the NO_2_ as the EWG; and the pair **293a** and **293b** with no substituent for comparison, to perform some photophysical studies (UV–vis absorption, fluorescence emission, quantum yield, and fluorescence lifetimes) related to fluorescence properties of these compounds. Compounds **293a**, **293b**, **295a**, and **295b** exhibited absorption bands ranging from 250 to 400 nm, while **301a** and **301b** showed new absorption bands red-shifted. The fluorescence quantum yield (Φ_f_) values for the pair **293a** and **293b** solutions are relatively larger than those of **293b** and **295b**. In the case of isomers bearing a nitro group, an inversion of the Φ was observed, with **301b** exhibiting a more considerable value than **301a**. Studies of fluorescence lifetimes by the TCSPC technique indicated that for solutions of compounds **295a** and **295b**, the τ_1_ lifetimes are relatively longer than for **293a** and **293b** solutions, whereas the lifetimes for the solutions of compound **301a** and **301b** were shorter.

Although the reaction developed has proved efficient in promoting the C–H/N–H annulation of lapimidazole, the high-cost catalyst [RhCp*Cl_2_]_2_/AgOAc system may limit the applicability of this synthesis. In this regard, the same research group developed a similar methodology using a [RuCl_2_(*p*-cymene)]_2_ catalyst instead of a [RhCp*Cl_2_]_2_ catalyst (Scheme 32A).^158^ In this new approach, the authors described two methods using Cu­(OAc)_2_·H_2_O (1 equiv) as oxidant and methanol as solvent: method A) [RuCl_2_(*p*-cymene)]_2_ (5 mol %)/AgSbF_6_ (1.5 equiv) at 120 °C and method B) [RuCl_2_(*p*-cymene)]_2_ (10 mol %)/AgSbF_6_ (2.0 equiv) at 110 °C. These protocols were applied to different lapimidazole bearing EDG and EWG. Similar to the previous work (Scheme 31),^139^ the main product displayed the installed phenyl groups at the benzo side, and the secondary product formed via annulation in the aliphatic site for both methods. The general yields were lower in this case when using [RuCl_2_(*p*-cymene)]_2_/AgOAc system instead of [RhCp*Cl_2_]_2_/AgOAc system. Compounds **293a**, **293b**, **294b**, **301a**, **299a**, and **308a** had their structure confirmed by X-ray diffraction studies, further supporting the regioselectivity. Protocol B was also applied using diaryl and dialkyl alkynes, affording products **309**–**314** (Scheme 32B).

**Scheme 32:**
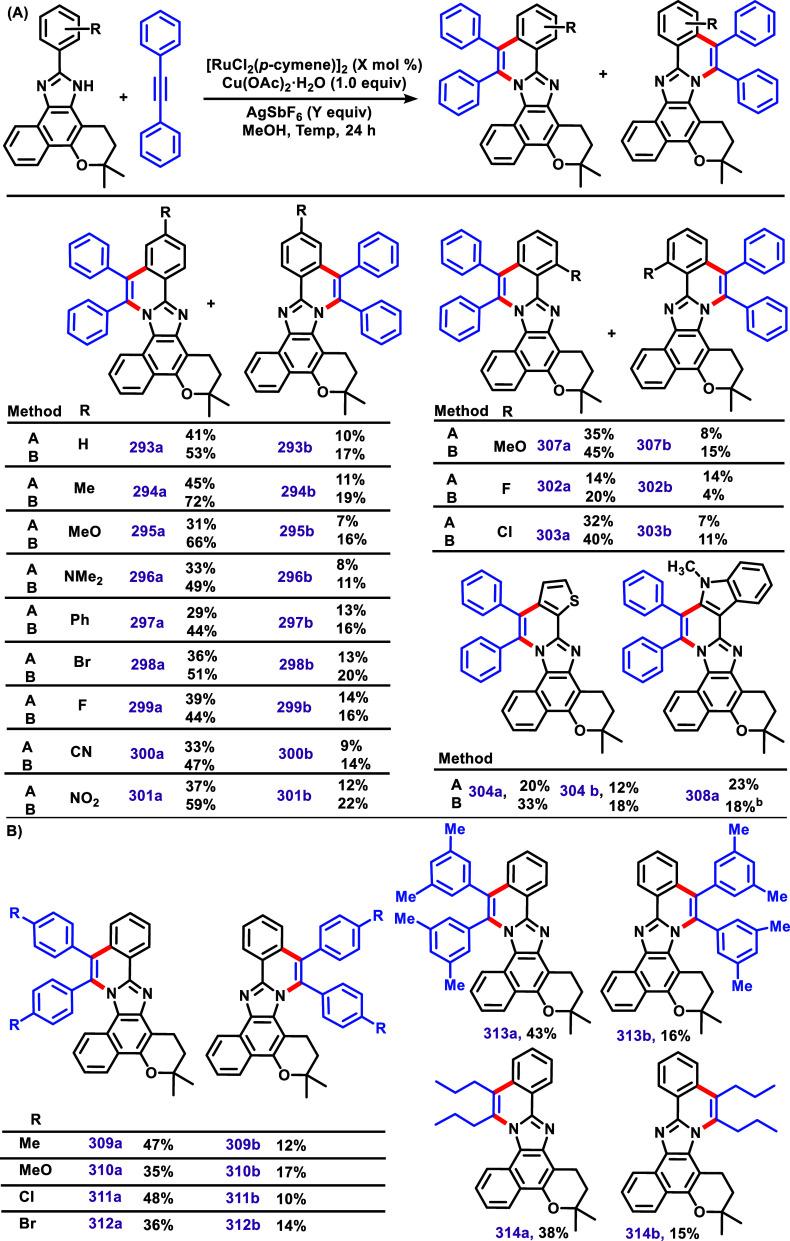
(A) C–H/N–H Annulation of Aryl-Lapimidazole Using the Two Methods: Method A) [RuCl_2_(*p*-cymene)]_2_ (5 mol %)/AgSbF_6_ (1.5 equiv) at 120 °C and Method B) [RuCl_2_(*p*-Cymene)]_2_ (10 mol %)/AgSbF_6_ (2.0 equiv) at 110 °C. (B) Method B Applied to Promote C–H/N–H Annulation of Aryl-Lapimidazole Using Diverses Alkynes.^158^

The authors also applied method B to similar nor-lapimidazole derivatives, bearing a furane-type ring instead of a pyran-type ring found in lapimidazole (Scheme 33). Curiously, in this case, the smaller furan-type ring induced the C–H/N–H annulation in the furane-type ring side instead of the benzo side. Structures **293a** and **315a** were confirmed by X-ray diffraction studies, supporting the regioselectivity.

**Scheme 33:**
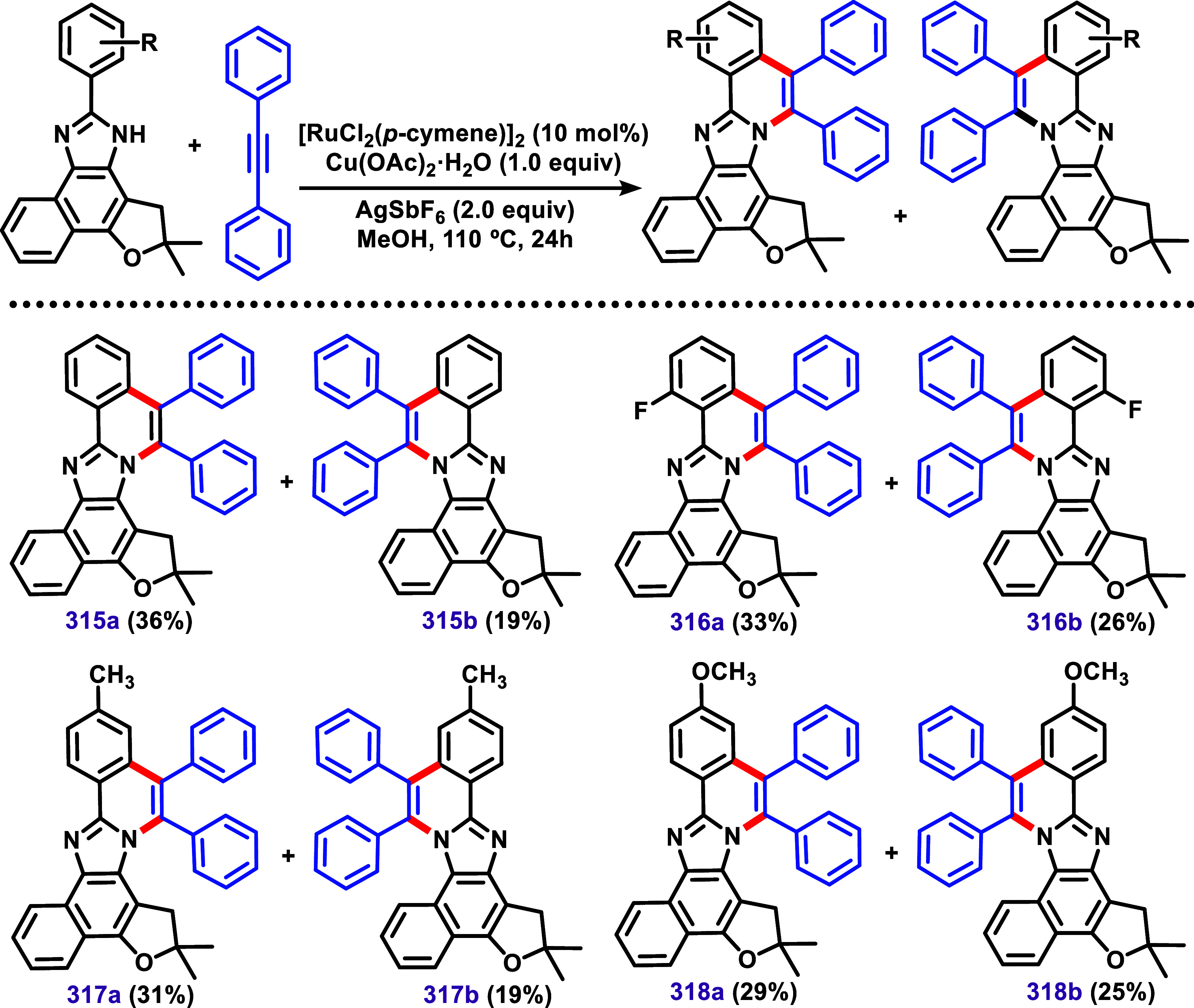
C–H/N–H Annulation of Aryl-Nor-Lapimidazole Using [RuCl_2_(*p*-cymene)]_2_ (10 mol %)/AgSBF_6_ (2.0 equiv) System.^158^

The possible mechanism of this reaction was studied using DFT, and the results indicated that the preference for the formation of compound **293a** over its isomer **293b** may be attributed to a larger steric hindrance at the aliphatic site of the lapimidazole, which increases the energy of the intermediates and transition states.

The authors also described complementary experiments on the optical properties of compounds **293a**, **293b**, **295a**, **295b**, **301a**, and **301b** via UV–vis absorption, time-resolved fluorescence, and steady-state fluorescence emission. According to the authors, the emission and absorption spectra, as well as the corresponding decay curves, for these compounds depend on the nature of the substituent group. This result demonstrates that new conformational states were established, resulting in distinct optical features.

Later, the authors investigated the photophysical properties, including time-resolved and steady-state fluorescence measurements of compounds **293a** and **293b** on drop-cast films, powders, and diluted solutions.^159^ In this study, TCSPC measurements showed that the temporal decay curve depends on how the samples are treated during the measurements. In addition, theoretical calculations have shown that even small structural modifications in molecules can significantly impact their conformational dynamics, electronic spectra, and overall electronic behavior and stability.

## 7 Conclusion

The development of synthetic methodologies based on C–H/N–H annulation of imidazoles has emerged as a key strategy for obtaining highly conjugated and fluorescent systems. Constructing these structures using other synthetic methods would be extremely labor-intensive. The ongoing efforts to improve reaction conditions highlight the significance of these new protocols. This review discussed how rhodium, ruthenium, cobalt, palladium, and nickel catalysts have been applied to achieve benzo­[4,5]­imidazo­[2,1-*a*]­isoquinoline via C–H/N–H annulation of imidazoles. These methodologies have been modified to become increasingly efficient, primarily by reducing costs and improving the availability of catalysts. For instance, catalysts based on nickel and ruthenium have become more sought after. Additionally, the advent of electrocatalysis has made it possible to replace metallic additives, such as copper salts, further enhancing the appeal of this strategy. In the field of synthetic methodology development, there are still few examples of the use of nonsymmetrical imidazoles and alkynes. This represents an interesting area for further exploration, as such structural asymmetry, combined with electronic and steric effects, could lead to structural diversification, as discussed in terms of molecular rearrangements and the selective formation of a single product.

Despite the progress made, there is still much to be achieved, including further studies on nonsymmetrical substrates and reagents to develop conditions that favor the formation of specific products. There is also a need to search for cheaper and more efficient catalysts, as well as milder reaction conditions, among other improvements. An important aspect that remains underexplored is the potential applications of these products, particularly concerning their optical properties. Few studies have focused on using annulated products in optical detection devices, OLED systems, electronic conductivity, or as biomarkers, among other potential applications.
